# Supplementary material for: Influence of aggregation on benthic coral reef spatio-temporal dynamics
Source: R Soc Open Sci. 2019 Feb 20;6(2):181703. doi: 10.1098/rsos.181703 (PMC6408412; doi:10.1098/rsos.181703)
Supplement: Spatial pattern evolution & 2D power spectra; FNN analysis - 3 dimensions resolves dynamics; Spatio-temporal forecasting for additional pathways; Pathways in varying herbivore scenarios; Incorporating diffusion into model [file rsos181703supp1.pdf]

Supplementary materials

Contents

ESM file 1
Title: Spatial pattern evolution & 2D power spectra
Caption: Example of pattern evolution & 2D power spectra showing duration of initial condition.
ESM file 2
Title: FNN analysis - 3 dimensions resolves dynamics
Caption: Fraction of false nearest neighbors decreases to near zero at three embedding dimensions.
ESM file 3
Title: Spatio-temporal forecasting for additional pathways
Caption: Forecasting results consistently shows nonlinear dynamics in transient stages of pathways.
ESM file 4
Title: Pathways in varying herbivore scenarios
Caption: Pathways in herbivore-limited scenarios exhibit amplified aggregation delay.
ESM file 5
Title: Incorporating diffusion into model
Caption: Description of methods for incorporating CO diffusion into the model that enables clumping in the attractor, but also depresses the steady-state CO fractional cover.
ESM file 6
Title: Pathways with stochastic disturbance (storm events)
Caption: Stochastic disturbance events did not significantly influence the delay effect of aggregation on duration time towards attractors in herbivore-abundant and herbivore-limited reefs.
ESM file 7
Title: Pathways initialized with small competitively inferior colonies
Caption: Initializing with smaller competitively inferior colonies from high- and low-coral cover scenarios shows faster arrivals at attractors, but with aggregation delay still present.
ESM file 8
Title: Pathway stages (presence of transient) robust to grazing variations
Caption: Transient stage present within pathways shows effect of aggregation on reef evolution is robust irrespective of grazing algorithm.
ESM file 9
Title: Data – Fig 3
Caption: Calculated time scales and durations of phases by aggregation level.

ESM file 1

Spatial pattern evolution

To explore the evolution of the initial spatial pattern configuration within each of the four stages of the pathway, two-dimensional spatial power spectra of CO distribution were calculated on a detrended binary CO cellular array. The power spectral density is characterized by peaks associated with spacing of the initial aggregation (10m) in the repelling stage, which dissipates in the transient stage despite remnants of the initial pattern remaining visible into the attracting stage and finally disappearing in the attractor (Fig. S1).

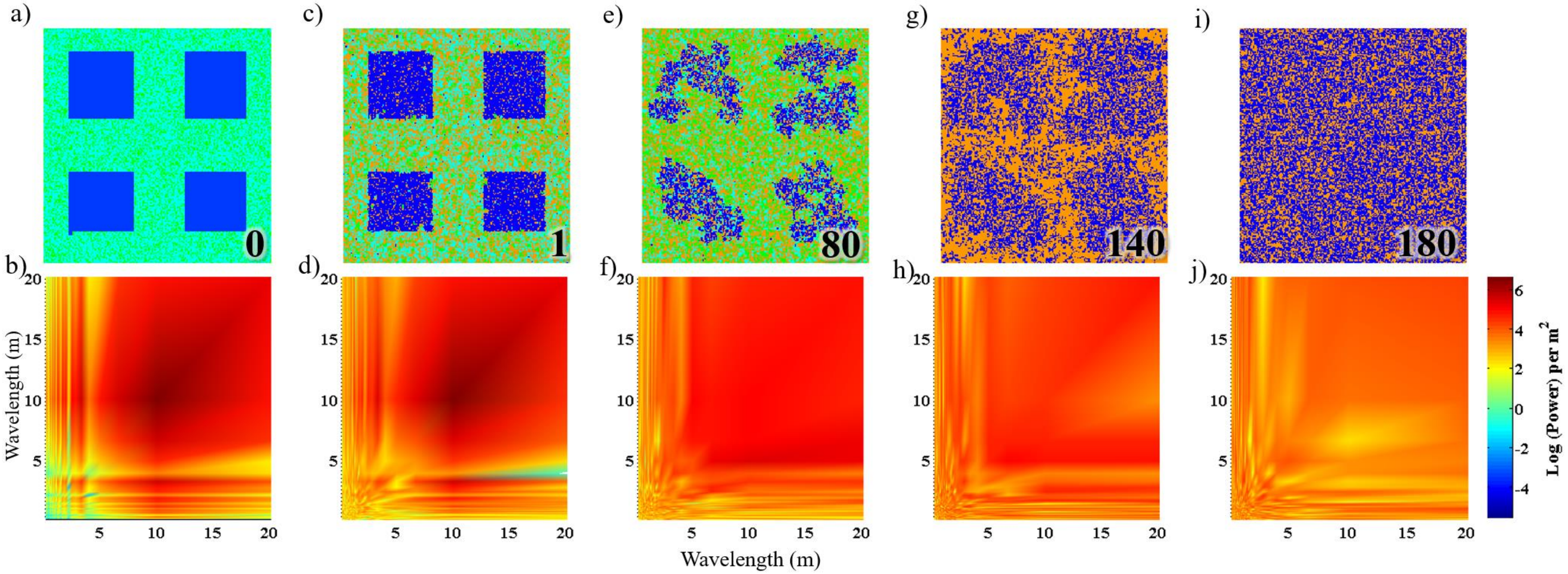

Figure S1: Snapshots of model lattice evolution (top row) and corresponding two-dimensional power spectra (bottom row) of detrended binary CO cellular arrays for an initial condition with four CO clumps at a) & b) initial state; year 0, c) & d) repelling stage; year 1, e) & f) transient stage; year 80, g) & h) attracting stage, year 140, i) & j) attractor stage, year 180. Spike (10 m) associated with spacing of initial aggregation pattern dissipates in transient stage.

ESM file 2

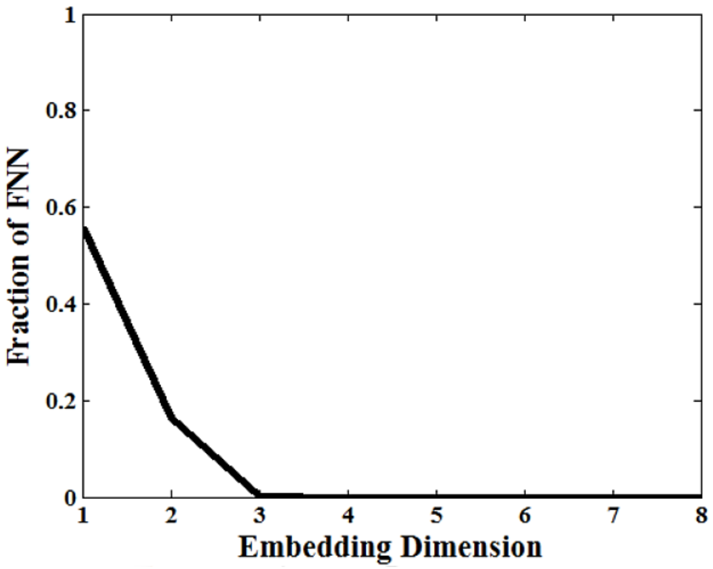

Figure S2: Fraction of false nearest neighbors vs embedding dimension from phase space reconstruction of CO fractional cover 250-year time series in the attractor of the clumped initial condition (aggregation = 0.99; lag = 5 time steps). False nearest neighbors decrease to near zero at three embedding dimensions, indicating that three dimensions is sufficient to resolve system dynamics.

ESM file 3

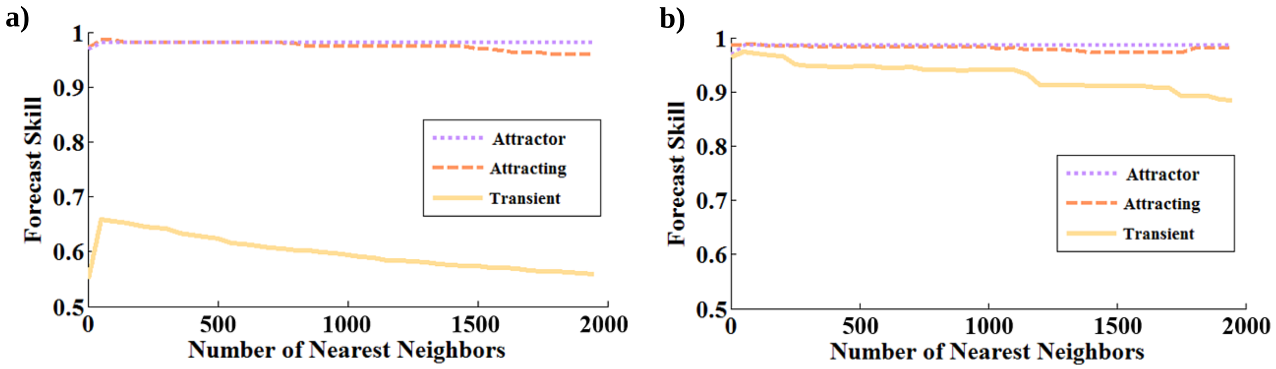

Figure S3: Nonlinear spatio-temporal forecasting for pathways with initial aggregation (a) 0.88 and (b) 0.66. Nonlinear dynamics dominated transient stage for high values of initial aggregation, but nonlinearity was only marginally present in (b) because the transient stage in this pathway was short and interactions weak.

ESM file 4

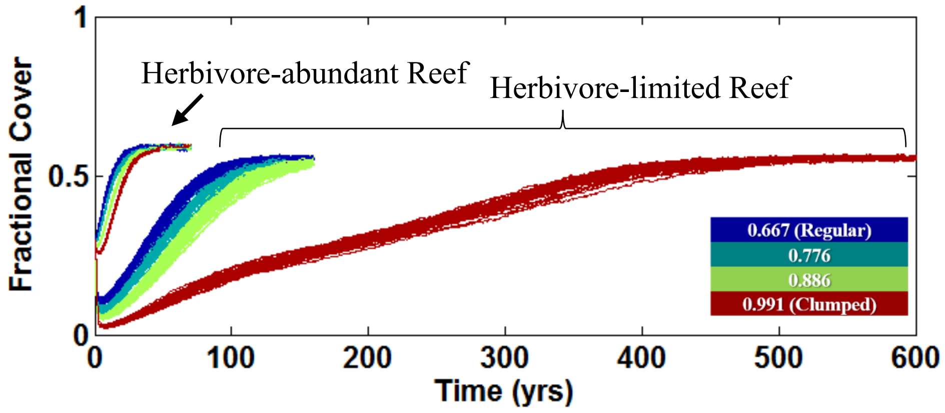

Figure S4: CO fractional cover vs. time for four values of initial aggregation (see color bar) for an herbivore-abundant reef (24% of lattice grazed per time step) and a herbivore-limited reef (4% of lattice grazed per time step). n = 50. Herbivore-limited reefs greatly amplify the effect of aggregation on the time duration to arrive at the attractor. (Pathways used to generate figure 4 in paper)

ESM file 5

Incorporating Coral Diffusion into the Model

In the model, the dynamics of coral (slow) and algae (fast) fractional cover are scale-separated, but coral fractional cover and aggregation only becomes scale separated from coral growth processes when diffusional processes are included.

In the model, the lack of scale separation is indicated by near coincidence of the cell-level CO growth time scale (~10 years) and the typical time scales for the reefscape CO fractional cover dynamics (~8 years) (i.e. the ratio of decay time scales of fast- and slow-scale processes is near unity). Thus, the model was modified to include CO diffusion, which is dissipative, in the following manner. A first order solution of the diffusion equation for CO was implemented on a separate cellular lattice and transferred to the model lattice tracking the mutually exclusive occurrence of the four functional groups in each cell under the following two conditions. (1) If the value of CO in a cell in the diffusion lattice goes from less than 0.5 to greater than 0.5, the value of that cell in the diffusion lattice is set to 1 and the corresponding cell of the model lattice is set to CO. (2) If the value of CO in a cell in the diffusion lattice goes from greater than 0.5 to less than 0.5, the value of that cell in the diffusion lattice was set to 0 and the corresponding cell of the model lattice was set to MA, TA or CCA, randomly choosing the type amongst the neighboring cells. This process is constrained by the requirement that the net change in CO cells on the model lattice is 0. Coral growth rate was increased from 0.1m/y to 0.2m/y, which is within the measured range for branching coral types (70), because of the effect of diffusion on steady-state coral fractional cover (see below).

The addition of diffusion increases the time scale of decay to the attractor from 3.43 years to 18.80 years over a diffusion constant range of 0 to 0.16, suggesting that, with diffusion, emergent CO clumps became self-organized and scale-separated from CO growth and mortality processes. Diffusion leads to increased CO clumpiness, but only slight increases in CO aggregation, because the aggregation increase is offset by a decrease in coral fractional cover (Fig 6a, b). Steady-state coral fractional cover decreases as the diffusion constant increases (Fig 6b). This reflects the effect of increased diffusion-driven aggregation on tipping the balance between CO mortality and growth (as described earlier with model experiments of increasingly clumped initial aggregation levels).

Supplementary References

70. Dullo W-C (2005) Coral growth and reef growth: a brief review. *Facies* 51(1–4):33–48.

ESM file 6

Effect of Stochastic Disturbance

The results shown in the article highlight the effect of aggregation on reefscape dynamics with competitively dominant coral colonies that exhibit increased coral survival and growth into space. The ability of large coral colonies to dominate reefscapes despite a degraded herbivore guild has been documented previously in Jamaica (71), where a coral dominated reef with a depressed herbivory guild transitioned into an algae dominated reef only upon experiencing a large hurricane and subsequent urchin disease outbreak. Here, we explore briefly how stochastic storm events affect the impact of aggregation on reefscape dynamics found in the main article. The model was modified so that storms occurred annually with probability 1/7. When a storm occurs, it kills 20% of whole coral colonies on the reefscape. Simulations were run for an herbivore-abundant (24% lattice grazed per time step) and herbivore-limited (4% lattice grazed per time step) reef for initial aggregation levels of 0.66 (regular) and 0.99 (clumped) (Fig. S6). Simulations were also run at moderate herbivory levels (12% lattice grazed per time step as in main paper) for the most clumped initialization to directly gage changes to the transient stage (Fig. S7).

In comparing CO fractional cover pathways in herbivore-abundant and herbivore-limited reef conditions, the effect of initial aggregation on coral fractional cover pathways remains significant, with regular initial aggregation configurations resulting in faster arrivals to the attractor (high grazer CO fractional cover attractor: 0.485 +/- 0.002 SD, low grazer CO cover attractor: < 0.001) (Fig. S6). For herbivore-abundant reefs, clumped initial configuration delays arrival to the attractor by a decade. For herbivore-limited reefs, the regular initial configuration prolongs coral persistence on the reef by more than a century.

In the model, we found that stochastic storm events in moderate-herbivory reef conditions result in a significantly prolonged transient stage that comes to dominate the duration of CO fractional cover pathways, thereby allowing CO to persist on the lattice for decades before it eventually becomes extinguished (Fig. S7). The transient stage under stormy conditions does not appear pronounced in the CO fractional cover pathway because storms occurring on faster time scales suppress the pathway from evolving into the attracting and attractor stages (unlike the pathway without storms). However, a comparison of snapshots of the reefscape lattice with (Fig. S7) and without storms (Fig. 2) shows the prolonged presence of the clumped configuration despite the loss of whole colonies during storm events. Based on these results, we can expect that predicted

recurrent and intensified storm disturbances (64), will result in the extended presence of the transient stage while CO persists on the reefscape, thus elongating the amount of time that the dynamics of the system are strongly nonlinear.

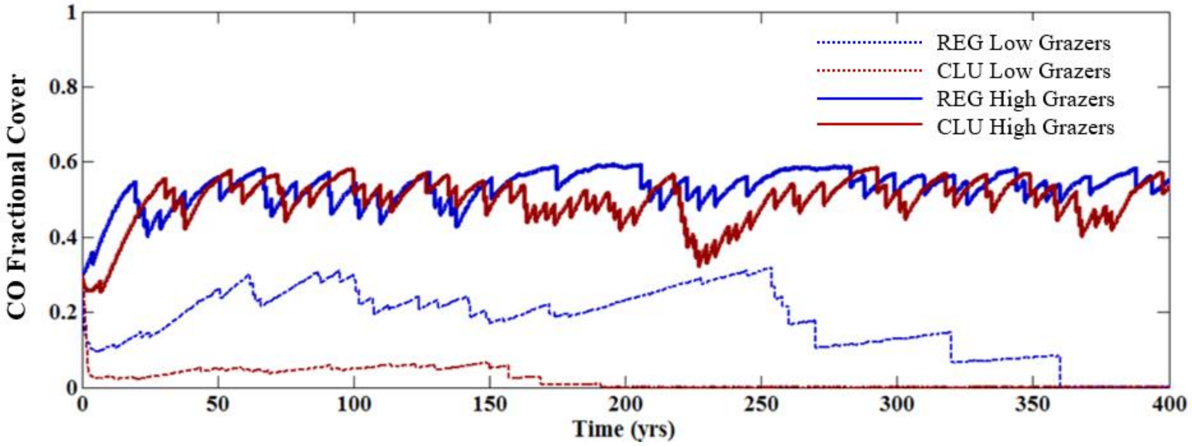

Figure S6: CO fractional cover vs. time for herbivore-abundant (heavy lines) and herbivore-limited (light lines) reefs starting from regular (0.71) and clumped (0.99) initial aggregations subject to storm disturbance. As shown with non-disturbance pathways (Fig S4), higher initial aggregation significantly prolongs arrival at the attractor in high grazer cases and prolongs persistence in low grazer cases, despite storms.

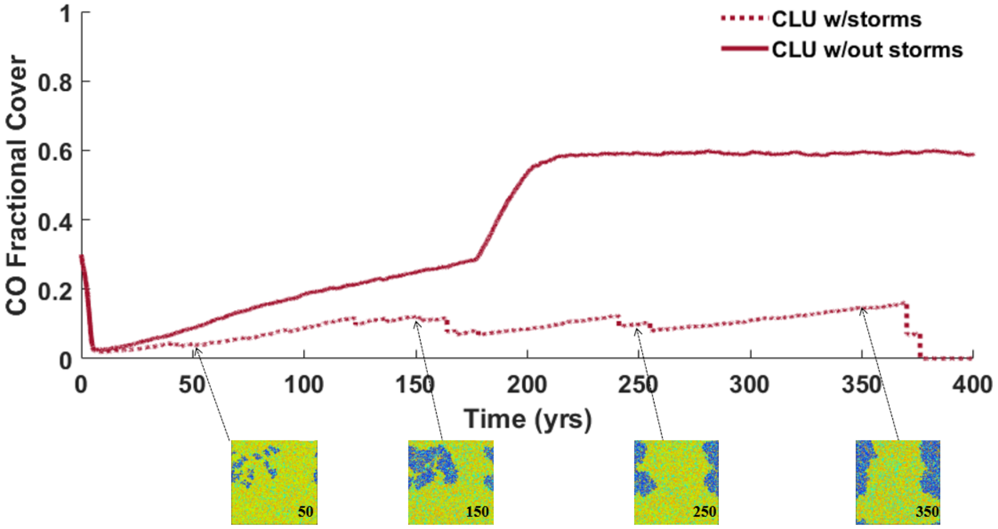

Figure S7: Under a storm disturbance regime (dotted line), the CO fractional cover pathway starting from a clumped initialization quickly becomes dominated by the transient stage characterized by strong nonlinear dynamics (i.e., coral-algae competition) that lasts for hundreds of years until coral drops out (unlike pathway w/out storms in which transient stage ends at ~175 yrs). Snapshots of the reefscape lattice at multiple points throughout the pathway. Blue: CO; green: MA; bluish green: TA; orange: CCA.

Supplementary References

71. Hughes T (1994) Catastrophes, phase shifts, and large-scale degradation of a Caribbean coral reef. *Science (80-)* 77(September 1994): 1547–1551.

ESM file 7

Effect of Small Colonies

The effect of initial aggregation on the more vulnerable, small, competitively inferior coral colonies was investigated because of its potential implications for management. Recruitment of small colonies into the adult population is necessary for the establishment of new competitively dominant coral in reefs (72). We simulated CO pathways initialized with regular and clumped aggregations for small coral colonies (4 contiguous cells) starting at fractional cover levels above (0.70) and below (0.30) the CO pathway attractors (herbivore-abundant: 0.586 +/- 0.002 SD; herbivore-limited: <0.001). Starting fractional cover above and below the attractor for herbivore-abundant reefs allowed for exploring recovery pathways (from below) and pathways that might be considered degrading rather than stabilizing (from above).

All small coral colony fractional cover pathways with clumped (red) initializations result in significant delays to the attractor in both herbivore-abundant and herbivore-limited scenarios (Fig. S8). In herbivore-limited reefs, clumping increases coral persistence by minimizing the number of coral borders that would be exposed to negative competitive interactions (Fig. S8a). In contrast, the apparent benefit of clumping becomes a disadvantage in herbivore-abundant reefs, where coral growth is constrained to the outer perimeter of the clump (until the clump is dissolved through mortality processes), thus producing a ten-year delay towards arrival at the steady-state attractor (Fig. S8b). Initializing coral pathways, above and below the attractor confirms that pathways eventually stabilize at the attractor determined by the parameters, including grazing level, independent of initial condition (basin of attraction was the entire state space). For herbivore-abundant reefs, starting coral fractional cover from above the attractor results in only a five-year delay towards steady-state for clumped initializations when compared to initializations below the attractor, challenging the expectation that the coral pathway would have stabilized much sooner (traceable to the constraint on growth by clumping). Also, for herbivore-abundant reefs, the clumped coral fractional cover pathway from above the attractor crossed the attractor level early (~year 3), which can be misinterpreted as an arrival at a steady-state attractor if not viewed through a dynamics lens. Connecting significant declines in coral cover to high levels of aggregation can aid management responses in the face of ‘unexpected’ declines.

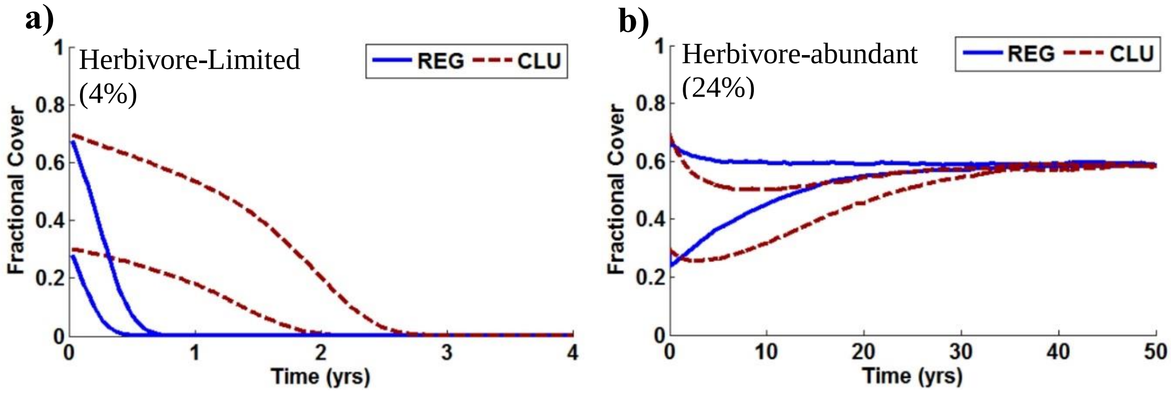

Figure S8: CO fractional cover vs. time for (a) herbivore-limited and (b) herbivore-abundant reefs for both regular (blue) and clumped (red) initial aggregations of small coral colonies. Clumping prolongs CO persistence for 1.5 years for herbivore-limited reefs and delayed arrival to the steady-state by ~10 years for herbivore-abundant reefs.

Supplementary References

72. Hughes T, et al. (2000) Supply-side ecology works both ways: the link between benthic adults, fecundity, and larval recruits. *Ecology* 81(8):2241–2249.

ESM file 8

Sensitivity to Grazing

Coupling benthic community to grazer population

To gauge the dependence of the emergent transient stage within pathways on the grazing parameterization, different variations of grazing on the lattice were explored. First, grazing is coupled to the benthic community through the incorporation of a herbivore biomass model (73), where herbivore biomass (H) changes according to:

$$\frac{dH}{dt} = \left(\frac{\mathrm{H}}{i_H + H}\right) \mathrm{g}\mu(\mathrm{MA} + \mathrm{TA}) - d_H H + l_H \quad , \qquad \text{(SE1)}$$

with the parameters are defined in table ST1. Herbivore biomass depends on the total amount of algae consumed minus a certain mortality rate plus larval recruitment into the population, which then determines the grazing level of benthic algae at each time step. A parameter sweep of combinations of minimum and maximum values was performed according to the values in table ST1 for a moderately aggregated initial configuration of 0.88, with the results summarized in figure S9. The transient stage also emerges in the pathways of this modified model, sometimes delaying the arrival at the attractor by more than one hundred years, but the marked drop of algae (and 'release' of CO arriving to attractor) disappears in most cases (refer to Fig.2 for comparison). MA fractional cover reduces to zero in the attractor only for case c, where grazing rate is maximized; variations of coral-algae coexistence attractors occur for the other cases. Notably, there is an additional delay effect of about 20-30 years to arrive at the attractor when compared to the uncoupled model, which could be tied to the fact that the level of herbivore grazing is coupled to the amount of algae on the lattice (meaning that if algae decreases, herbivory decreases thus prolonging the time it takes for algae to become extinguished).

Spatially constrained grazing in benthic-grazer coupled model

Targeted grazing of algae is included in the model by modifying grazer behavior from consumption of randomly chosen algae cells across the lattice to allowing grazers to ‘swim’ in one of four directions from a randomly selected cell until algae is found and consumed. As before, a parameter sweep was performed using the values in table ST1 and a moderately aggregated initial configuration of 0.88 (results summarized in Fig. S10). The presence of the transient stage within these pathways again emphasizes the robustness of the delay effect of aggregation on reef evolution, irrespective the type of grazing algorithm implemented.

Supplementary References

73. Melbourne-Thomas J, et al. (2011) A multi-scale biophysical model to inform regional management of coral reefs in the western Philippines and South China Sea. *Environ Model Softw* 26(1):66

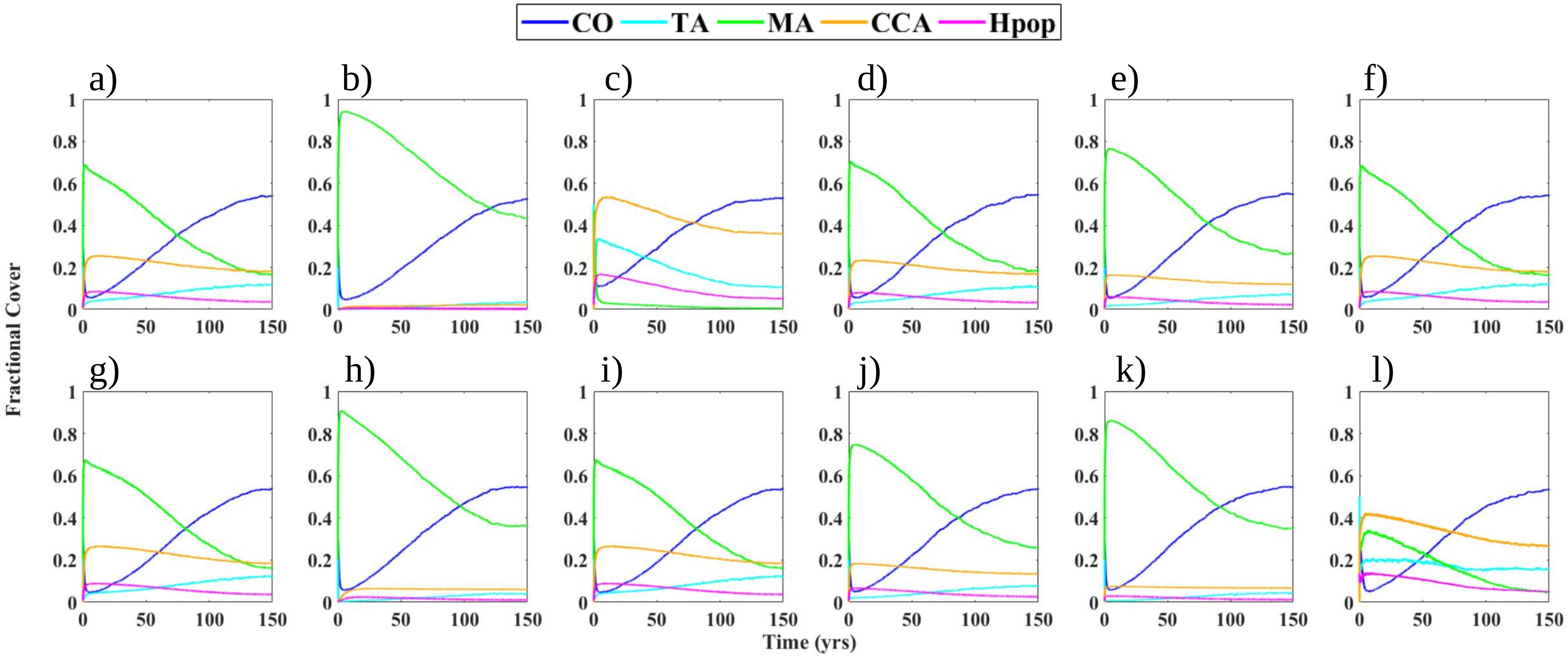

Figure S9: In each panel, fractional cover pathways through time for all functional types (CO = coral, TA = turf algae, MA = macroalgae, CCA = crustose coralline algae, Hpop = herbivores) for each set of parameters (in table ST1) for the coupled benthos-grazer model.

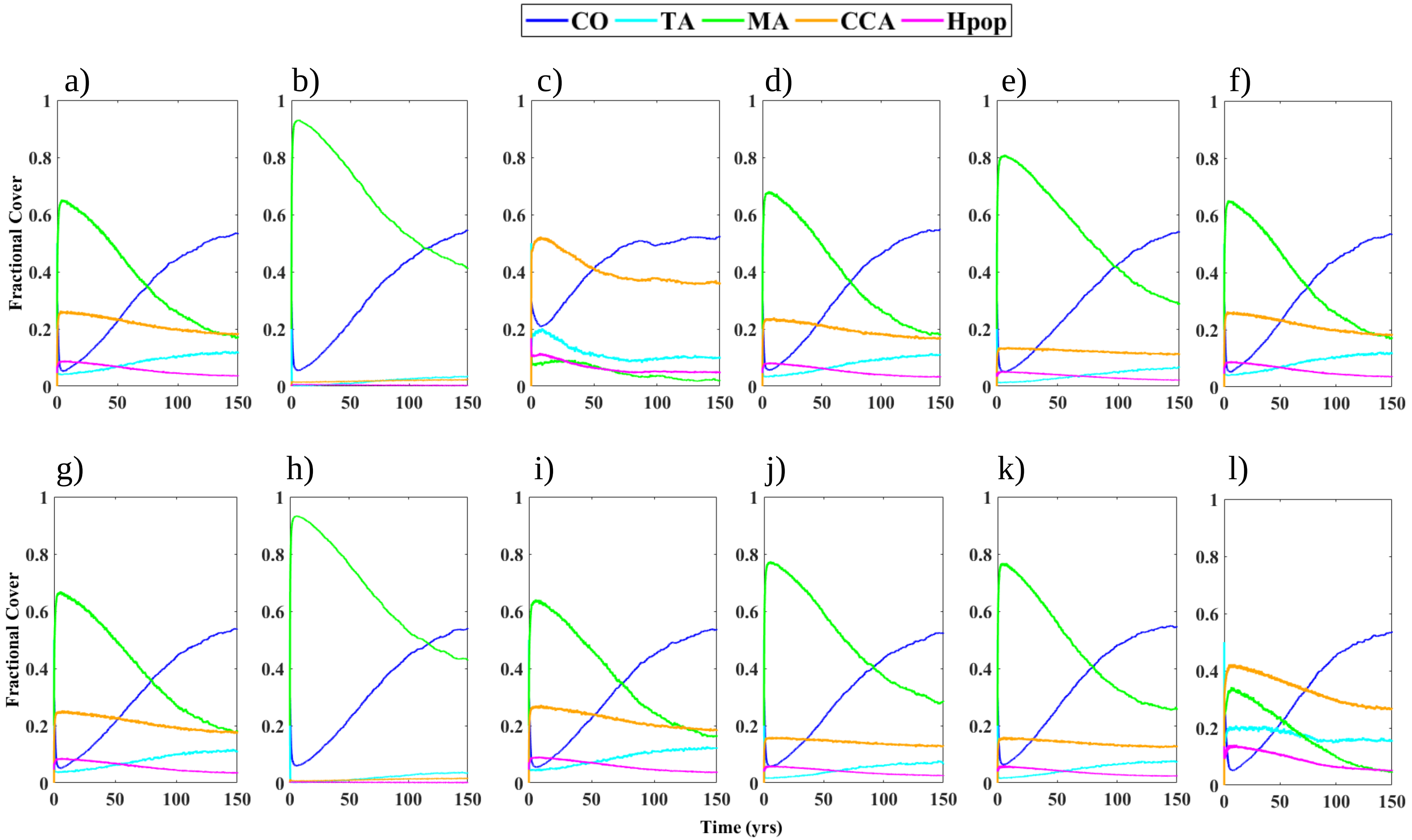

Figure S10: Dynamics of functional types for parameter sweep of coupled benthic-grazer model with targeted grazing. Grazing rate parameters used for each panel are in table ST1. CO = coral, TA = turf algae, MA = macroalgae, CCA = crustose coralline algae, Hpop = herbivores.

Table ST1: Parameter definitions and values for the herbivore biomass model to which the benthic community is coupled. Sets of parameter values correspond to results by letter label in Figures S9 and S10.

			figure panels											
parameters	interpretation	unit	a (min)	b	c	d	e	f	g	h (max)	i	j	k	l
i_H	algae accessibility to grazing	kg/km^2	3000	500000	3000	3000	3000	3000	3000	500000	3000	3000	3000	3000
g	grazing rate	/yr	5	5	15	5	5	5	5	15	5	5	5	15
ς_T	rate at which CCA to TA	/yr	2	2	2	20	2	2	2	20	2	2	20	20
μ	fish biomass from grazing	$kg/km^2/yr$	60	60	60	60	μ_{max}	60	60	μ_{max}	60	μ_{max}	μ_{max}	μ_{max}
d_H	fish mortality	/yr	dH_{min}	dH_{min}	dH_{min}	dH_{min}	dH_{min}	2	dH_{min}	2	2	2	2	2
ℓ_H	larvae production	/kg yr	5000	5000	5000	5000	5000	5000	7000	7000	7000	7000	7000	7000

*bolded/italicized: where threshold region occurs 1 = CO; 2 = MA

run names	Treat#	Trial (n)	type	agg_co	Lt Rep Limit	Rt Rep Limit	Attractor Lt Limit	Attractor Rt limit	Avg CO cells in att	Avg stdev	timesteps to att	reptimesc	trandur	atttimesc
names (3,1)= {'p30s3x3brf300REG03y50'};	1	1	1	0.66	2	17	336	1175	23625.14	10.366	1653	3.64906	7.98	7.32296
names (3,2)= {'p30s3x3brf300RAN03y50'};	2	1	1	0.711	2	17	397	1214	23414.31	30.260	1563	6.38779	9.50	7.47025
names (3,3)= {'p30s3x3brf300REG03clu31x31y85'};	3	1	1	0.721	2	17	1699	2660	23676.99	156.207	2873	6.32073	42.05	9.45422
names (3,4)= {'p30s3x3brf300REG03clu28x28y120'};	4	1	1	0.744	2	17	299	1020	23523.19	77.314	2079	6.31316	7.05	7.61481
names (3,5)= {'p30s3x3brf300REG03clu24x25y100'};	5	1	1	0.776	2	17	1905	3250	23509.53	89.128	3438	4.80964	47.20	8.97568
names (3,6)= {'p30s3x3brf300REG03clu18x19y100'};	6	1	1	0.831	2	17	2326	3271	23330.08	118.784	3557	6.12834	57.73	6.81574
names (3,7)= {'p30s3x3brf300REG03clu12x13y130'};	7	1	1	0.886	2	17	2778	3923	23483.06	34.770	4318	8.16236	69.03	8.30045
names (3,8)= {'p30s3x3brf300REG03clu9x10y120'};	8	1	1	0.913	2	17	3234	4010	23423.15	223.362	4310	7.82169	80.43	8.34681
names (3,9)= {'p30s3x3brf300REG03clu7x7y125'};	9	1	1	0.936	2	17	3681	4256	22861.59	294.004	4546	8.40238	91.60	7.89308
names (3,10)= {'p30s3x3brf300REG03clu4x4y150'};	10	1	1	0.963	2	17	4451	5439	23343.58	129.185	5570	11.91413	110.85	6.01504
names (3,11)= {'p30s3x3brf300REG03clu2x2y180'};	11	1	1	0.982	2	17	4922	6034	23543.63	13.964	6536	11.69996	122.63	7.99438
names (3,12)= {'p30s3x3brf300CLU03y250'};	12	1	1	0.991	2	17	7895	9249	23522.89	24.265	9635	12.39330	196.95	7.84804
names (4,1)= {'p30s3x3brf300REG04y50'};	1	2	1	0.66	2	18	368	1104	23430.22	36.898	1457	6.14542	8.75	7.59700
names (4,2)= {'p30s3x3brf300RAN04y50'};	2	2	1	0.711	2	18	410	997	23392.52	10.518	1558	6.28304	9.80	8.02715
names (4,3)= {'p30s3x3brf300REG04clu31x31y85'};	3	2	1	0.721	2	18	1633	2845	23507.76	5.514	3288	5.88589	40.38	9.31895
names (4,4)= {'p30s3x3brf300REG04clu28x28y120'};	4	2	1	0.744	2	18	1858	2801	23593.12	17.169	3439	5.60885	46.00	7.98415
names (4,5)= {'p30s3x3brf300REG04clu24x25y100'};	5	2	1	0.776	2	18	2144	3209	23411.34	193.519	3372	6.01060	53.15	8.06622
names (4,6)= {'p30s3x3brf300REG04clu18x19y100'};	6	2	1	0.831	2	18	2239	3195	23232.12	244.377	3385	7.21462	55.53	8.10244
names (4,7)= {'p30s3x3brf300REG04clu12x13y130'};	7	2	1	0.886	2	18	2548	4398	23520.19	138.355	3921	6.90399	63.25	8.16855
names (4,8)= {'p30s3x3brf300REG04clu9x10y120'};	8	2	1	0.913	2	18	3158	3991	23261.19	284.557	4196	9.13912	78.50	8.14691
names (4,9)= {'p30s3x3brf300REG04clu7x7y125'};	9	2	1	0.936	2	18	3548	4200	23219.85	355.546	4457	9.78940	88.25	8.91782
names (4,10)= {'p30s3x3brf300REG04clu4x4y150'};	10	2	1	0.963	2	18	3824	5309	23601.22	119.90753	5155	11.03050	95.15	6.20883
names (4,11)= {'p30s3x3brf300REG04clu2x2y180'};	11	2	1	0.982	2	18	5556	6431	23334.39	138.296	6734	11.19481	138.45	8.40307
names (4,12)= {'p30s3x3brf300CLU04y250'};	12	2	1	0.991	2	18	6495	7464	23557.15	75.104	8050	12.79129	161.93	9.35002
names (5,1)= {'p30s3x3brf300REG05y50'};	1	3	1	0.66	2	17	352	1142	23515.18	9.846	1635	5.76647	8.38	8.16485
names (5,2)= {'p30s3x3brf300RAN05y50'};	2	3	1	0.711	2	17	403	1345	23653.04	11.356	1685	3.73916	9.65	7.78303
names (5,3)= {'p30s3x3brf300REG05clu31x31y85'};	3	3	1	0.721	2	17	395	1968	23479.00	106.378	1523	6.90480	9.45	6.64060
names (5,4)= {'p30s3x3brf300REG05clu28x28y120'};	4	3	1	0.744	2	17	1945	3304	23568.68	35.967	3631	5.49587	48.20	8.26612
names (5,5)= {'p30s3x3brf300REG05clu24x25y100'};	5	3	1	0.776	2	17	1932	2884	23497.72	25.949	3395	6.04053	47.88	8.09887
names (5,6)= {'p30s3x3brf300REG05clu18x19y100'};	6	3	1	0.831	2	17	2401	3207	23205.84	212.123	3519	7.29426	59.60	9.15714
names (5,7)= {'p30s3x3brf300REG05clu12x13y130'};	7	3	1	0.886	2	17	2836	3802	23534.42	42.053	4274	7.51829	70.48	8.34146
names (5,8)= {'p30s3x3brf300REG05clu9x10y120'};	8	3	1	0.913	2	17	3370	4007	23063.72	398.506	4226	8.78007	83.83	8.05292
names (5,9)= {'p30s3x3brf300REG05clu7x7y125'};	9	3	1	0.936	2	17	3402	4187	23048.93	225.6141749	4498	9.54369	84.63	9.77527
names (5,10)= {'p30s3x3brf300REG05clu4x4y150'};	10	3	1	0.963	2	17	4302	5407	23469.62	81.495	5624	10.24907	107.13	8.03150
names (5,11)= {'p30s3x3brf300REG05clu2x2y180'};	11	3	1	0.982	2	17	5269	6490	23502.08	74.342	6683	11.60595	131.30	8.14923
names (5,12)= {'p30s3x3brf300CLU05y250'};	12	3	1	0.991	2	17	7891	9237	23443.38	27.112	9613	13.72540	196.85	6.21724
names (6,1)= {'p30s3x3brf300REG06y75'};	1	4	1	0.66	2	17	344	1358	23795.43	12.507	2984	6.17426	8.18	7.90143
names (6,2)= {'p30s3x3brf300RAN06y50'};	2	4	1	0.711	2	17	396	935	23480.12	40.251	1446	6.20450	9.48	8.39782
names (6,3)= {'p30s3x3brf300REG06clu31x31y85'};	3	4	1	0.721	2	17	1793	2674	23428.20	149.077	2914	6.30837	44.40	9.05709
names (6,4)= {'p30s3x3brf300REG06clu28x28y120'};	4	4	1	0.744	2	17	1707	3530	23626.03	6.024	4296	5.98365	42.25	6.87746
names (6,5)= {'p30s3x3brf300REG06clu24x25y100'};	5	4	1	0.776	2	17	2169	3242	23408.74	166.277	3407	5.77277	53.80	7.79027
names (6,6)= {'p30s3x3brf300REG06clu18x19y100'};	6	4	1	0.831	2	17	2147	3276	23378.97	103.702	3481	6.51446	53.25	6.64618
names (6,7)= {'p30s3x3brf300REG06clu12x13y130'};	7	4	1	0.886	2	17	2526	3983	23585.96	31.534	4267	6.94898	62.73	6.96932
names (6,8)= {'p30s3x3brf300REG06clu9x10y120'};	8	4	1	0.913	2	17	3219	4080	23082.13	134.9175106	4372	8.02589	80.05	6.70151
names (6,9)= {'p30s3x3brf300REG06clu7x7y125'};	9	4	1	0.936	2	17	3439	4264	23187.65	142.721	4577	8.96488	85.55	7.67721
names (6,10)= {'p30s3x3brf300REG06clu4x4y150'};	10	4	1	0.963	2	17	4001	5358	23458.04	28.923	5681	10.92100	99.60	9.34798
names (6,11)= {'p30s3x3brf300REG06clu2x2y180'};	11	4	1	0.982	2	17	5683	6401	23219.89	292.184	6642	11.55928	141.65	8.35390
names (6,12)= {'p30s3x3brf300CLU06y250'};	12	4	1	0.991	2	17	7133	8227	23502.59	27.003	8740	11.85389	177.90	6.42779
names (7,1)= {'p30s3x3brf300REG07y50'};	1	5	1	0.66	2	13	364	1244	23388.23	13.380	1563	5.71596	8.78	6.73747
names (7,2)= {'p30s3x3brf300RAN07y50'};	2	5	1	0.711	2	13	389	1567	23531.37	21.210	1662	7.03127	9.40	5.12528
names (7,3)= {'p30s3x3brf300REG07clu31x31y85'};	3	5	1	0.721	2	13	402	1440	23513.11	5.798	3389	7.15140	9.73	6.82901
names (7,4)= {'p30s3x3brf300REG07clu28x28y120'};	4	5	1	0.744	2	13	1953	3429	23540.90	58.043	3524	5.36954	48.50	7.53103
names (7,5)= {'p30s3x3brf300REG07clu24x25y100'};	5	5	1	0.776	2	13	1931	3219	23466.82	176.827	3219	6.42611	47.95	6.39391
names (7,6)= {'p30s3x3brf300REG07clu18x19y100'};	6	5	1	0.831	2	13	2500	3277	23092.82	154.693	3583	6.17257	62.18	6.85461
names (7,7)= {'p30s3x3brf300REG07clu12x13y130'};	7	5	1	0.886	2	13	2574	4009	23751.80	34.62332446	4418	8.23073	64.03	10.05635
names (7,8)= {'p30s3x3brf300REG07clu9x10y120'};	8	5	1	0.913	2	13	3152	4037	23197.27	36.625	4529	8.22151	78.48	8.12265
names (7,9)= {'p30s3x3brf300REG07clu7x7y125'};	9	5	1	0.936	2	13	3285	4270	23310.79	107.619	4557	10.50391	81.80	7.69204
names (7,10)= {'p30s3x3brf300REG07clu4x4y150'};	10	5	1	0.963	2	13	4029	5359	23413.96	112.667	5341	10.42174	100.40	6.23748
names (7,11)= {'p30s3x3brf300REG07clu2x2y180'};	11	5	1	0.982	2	13	5957	6422	22626.53	528.287	6635	11.24690	148.60	8.37040
names (7,12)= {'p30s3x3brf300CLU07y250'};	12	5	1	0.991	2	13	8494	9161	23095.14	259.805	9484	12.17990	212.03	7.23676
names (8,1)= {'p30s3x3brf300REG08y50'};	1	6	1	0.66	2	16	350	1475	23532.62	16.322	1805	6.01721	8.35	8.34824
names (8,2)= {'p30s3x3brf300RAN08y50'};	2	6	1	0.711	2	16	398	1056	23653.35	25.923	1494	7.11123	9.55	7.95502
names (8,3)= {'p30s3x3brf300REG08clu31x31y85'};	3	6	1	0.721	2	16	1830	2695	23194.37	126.613	2886	5.83505	45.35	7.90449
names (8,4)= {'p30s3x3brf300REG08clu28x28y120'};	4	6	1	0.744	2	16	1752	2870	23592.73	84.957	3144	5.64395	43.40	7.40038
names (8,5)= {'p30s3x3brf300REG08clu24x25y100'};	5	6	1	0.776	2	16	2096	3223	23494.34	24.085	3718	6.00661	52.00	8.54393
names (8,6)= {'p30s3x3brf300REG08clu18x19y100'};	6	6	1	0.831	2	16	2200	3252	23452.16	89.28160256	3556	6.46763	54.60	7.71980
names (8,7)= {'p30s3x3brf300REG08clu12x13y130'};	7	6	1	0.886	2	16	2993	4234	23520.52	0.236	4988	6.91139	74.43	8.44874
names (8,8)= {'p30s3x3brf300REG08clu9x10y120'};	8	6	1	0.913	2	16	2924	4008	23442.93	23.040	4526	8.15992	72.70	8.32198
names (8,9)= {'p30s3x3brf300REG08clu7x7y125'};	9	6	1	0.936	2	16	3379	4251	23353.76	17.264	4783	9.30908	84.08	6.18413
names (8,10)= {'p30s3x3brf300REG08clu4x4y150'};	10	6	1	0.963	2	16	4143	5416	23325.27	5.109	5829	10.29072	103.18	6.38018
names (8,11)= {'p30s3x3brf300REG08clu2x2y180'};	11	6	1	0.982	2	16	5624	6377	23426.62	200.519	6738	11.46138	140.20	9.80668
names (8,12)= {'p30s3x3brf300CLU08y250'};	12	6	1	0.991	2	16	7013	8111	23479.51	43.013	8602	12.63984	174.93	8.16501
names (9,1)= {'p30s3x3brf300REG09y50'};	1	7	1	0.66	2	17	358	1333	23576.39	113.524	1421	3.38437	8.53	7.61352
names (9,2)= {'p30s3x3brf300RAN09y50'};	2	7	1	0.711	2	17	391	1389	23458.14	154.332	1384	7.37432	9.35	7.30815
names (9,3)= {'p30s3x3brf300REG09clu31x31y85'};	3	7	1	0.721	2	17	1650	2685	23459.78	112.108	2888	6.28408	40.83	8.97073
names (9,4)= {'p30s3x3brf300REG09clu28x28y120'};	4	7	1	0.744	2	17	1822	3890	23503.73	77.759	3725	5.74027	45.13	9.40866
names (9,5)= {'p30s3x3brf300REG09clu24x25y100'};	5	7	1	0.776	2	17	1998	3186	23606.10	0.496	3833	6.94046	49.53	7.23089
names (9,6)= {'p30s3x3brf300REG09clu18x19y100'};	6	7	1	0.831	2	17	2478	3277	23253.95	162.973	3564	6.68258	61.53	7.40655
names (9,7)= {'p30s3x3brf300REG09clu12x13y130'};	7	7	1	0.886	2	17	2792	3970	23467.26	19.030	4449	7.05587	69.38	8.84011
names (9,8)= {'p30s3x3brf300REG09clu9x10y120'};	8	7	1	0.913	2	17	3160	4006	23397.21	35.410	4549	8.30595	78.58	8.90035
names (9,9)= {'p30s3x3brf300REG09clu7x7y125'};	9	7	1	0.936	2	17	3069	4121	23430.56	4.897	4807	9.86647	76.30	7.56560
names (9,10)= {'p30s3x3brf300REG09clu4x4y150'};	10	7	1	0.963	2	17	4267	5445	23308.90	23.093	5747	10.44857	106.25	6.76903
names (9,11)= {'p30s3x3brf300REG09clu2x2y180'};	11	7	1	0.982	2	17	5144	6494	23497.46	33.632	6737	12.18279	128.18	6.79755
names (9,12)= {'p30s3x3brf300CLU09y250'};	12	7	1	0.991	2	17	7116	8340	23599.85	43.692	8741	12.20995	177.48	7.35791
names (10,1)= {'p30s3x3brf300REG10y50'};	1	8	1	0.66	2	16	335	1186	23520.58	113.072	1472	7.14031	7.98	8.85871
names (10,2)= {'p30s3x3brf300RAN10y50'};	2	8	1	0.711	2	16	394	955	23471.51	7.427	1710	6.71443	9.45	9.07802
names (10,3)= {'p30s3x3brf300REG10clu31x31y85'};	3	8	1	0.721	2	16	1625	2548	23400.80	103.254	2859	6.67194	40.23	9.39873
names (10,4)= {'p30s3x3brf300REG10clu28x28y120'};	4	8	1	0.744	2	16	1820	3350	23677.26	31.052	3621	5.84812	45.10	8.76959
names (10,5)= {'p30s3x3brf300REG10clu24x25y100'};	5	8	1	0.776	2	16	1813	2842	23555.35	27.615	3353	6.69234	44.93	8.96210
names (10,6)= {'p30s3x3brf300REG10clu18x19y100'};	6	8	1	0.831	2	16	2180	3224	23297.10	4.572	3848	6.73682	54.10	7.87034
names (10,7)= {'p30s3x3brf300REG10clu12x13y130'};	7	8	1	0.886	2	16	2913	4174	23574.16	3.245	4798	7.92261	72.43	8.51916
names (10,8)= {'p30s3x3brf300REG10clu9x10y120'};	8	8	1	0.913	2	16	2866	3952	23692.86	5.381	4634	8.58238	71.25	9.19062
names (10,9)= {'p30s3x3brf300REG10clu7x7y125'};	9	8	1	0.936	2	16	3386	4189	23282.38	229.384	4483	9.87331	84.25	9.27696
names (10,10)= {'p30s3x3brf300REG10clu4x4y150'};	10	8	1	0.963	2	16	3835	4976	23521.04	17.921	5350	11.08836	95.48	7.04791
names (10,11)= {'p30s3x3brf300REG10clu2x2y180'};	11	8	1	0.982	2	16	5497	6394	23457.09	158.606	6693	11.74211	137.03	9.33785
names (10,12)= {'p30s3x3brf300CLU10y250'};	12	8	1	0.991	2	16	7661	9023	23648.82	0.369	9804	12.06798	191.13	6.86852
names (11,1)= {'p30s3x3brf300REG11y50'};	1	9	1	0.66	2	16	353	1215	23503.92	39.528	1480	5.81570	8.43	7.04757
names (11,2)= {'p30s3x3brf300RAN11y50'};	2	9	1	0.711	2	16	366	1139	23268.60	48.597	1389	6.46771	8.75	6.78228
names (11,3)= {'p30s3x3brf300REG11clu31x31y85'};	3	9	1	0.721	2	16	1881	2945	23646.44	91.321	3087	5.63308	46.63	9.27962
names (11,4)= {'p30s3x3brf300REG11clu28x28y120'};	4	9	1	0.744	2	16	1860	4151	23570.34	34.160	3584	5.15389	46.10	8.83703
names (11,5)= {'p30s3x3brf300REG11clu24x25y100'};	5	9	1	0.776	2	16	2020	3248	23515.20	121.565	3425	7.11673	50.10	9.12134
names (11,6)= {'p30s3x3brf300REG11clu18x19y100'};	6	9	1	0.831	2	16	2292	3236	23449.67	134.875	3555	6.71124	56.90	8.98782
names (11,7)= {'p30s3x3brf300REG11clu12x13y130'};	7	9	1	0.886	2	16	2858	4137	23656.58	8.831	4699	7.30994	71.05	8.55877
names (11,8)= {'p30s3x3brf300REG11clu9x10y120'};	8	9	1	0.913	2	16	2908	4016	23531.06	32.114	4493	8.24483	72.30	8.70029
names (11,9)= {'p30s3x3brf300REG11clu7x7y125'};	9	9	1	0.936	2	16	3181	4157	23389.58	243.519	4338	9.41768	79.13	9.02526
names (11,10)= {'p30s3x3brf300REG11clu4x4y150'};	10	9	1	0.963	2	16	3832	5305	23556.83	94.867	5252	10.13248	95.40	7.41932
names (11,11)= {'p30s3x3brf300REG11clu2x2y180'};	11	9	1	0.982	2	16	5848	6458	22800.22	276.766	6716	11.66810	145.80	7.42319
names (11,12)= {'p30s3x3brf300CLU11y250'};	12	9	1	0.991	2	16	6967	8614	23533.33	13.260	8961	13.32790	173.78	7.92417
names (12,1)= {'p30s3x3brf300REG12y50'};	1	10	1	0.66	2	18	347	1353	23462.43	1.017	1847	6.10526	8.23	6.43300
names (12,2)= {'p30s3x3brf300RAN12y50'};	2	10	1	0.711	2	18	384	1398	23396.43	92.761	1463	7.10429	9.15	7.07921
names (12,3)= {'p30s3x3brf300REG12clu31x31y85'};	3	10	1	0.721	2	18	411	1445	23513.43	93.935	1575	6.39867	9.83	8.18150
names (12,4)= {'p30s3x3brf300REG12clu28x28y120'};	4	10	1	0.744	2	18	314	1056	23455.92	7.452	4786	6.30718	7.40	7.54466
names (12,5)= {'p30s3x3brf300REG12clu24x25y100'};	5	10	1	0.776	2	18	1992	3295	23568.85	89.741	3394	6.63832	49.35	6.37965
names (12,6)= {'p30s3x3brf300REG12clu18x19y100'};	6	10	1	0.831	2	18	2087	3259	23595.91	87.227	3525	6.73106	51.73	8.24816
names (12,7)= {'p30s3x3brf300REG12clu12x13y130'};	7	10	1	0.886	2	18	2861	3886	23583.85	8.673	4539	7.21465	71.08	8.63564
names (12,8)= {'p30s3x3brf300REG12clu9x10y120'};	8	10	1	0.913	2	18	3010	3951	23520.45	214.344	4214	7.96138	74.80	9.18559
names (12,9)= {'p30s3x3brf300REG12clu7x7y125'};	9	10	1	0.936	2	18	3250	4222	23436.52	10.869	4803	9.62950	80.80	7.37891
names (12,10)= {'p30s3x3brf300REG12clu4x4y150'};	10	10	1	0.963	2	18	4198	5324	23428.50	176.802	5420	9.91402	104.50	7.69014
names (12,11)= {'p30s3x3brf300REG12clu2x2y180'};	11	10	1	0.982	2	18	5224	6442	23465.01	165.824	6423	11.64370	130.15	5.50026
names (12,12)= {'p30s3x3brf300CLU12y250'};	12	10	1	0.991	2	18	8352	9175	23325.44	171.871	9499	12.79129	208.35	6.45313
names (13,1)= {'p30s3x3brf300REG13y50'};	1	11	1	0.66	2	17	354	1437	23301.97	42.178	1607	5.76031	8.43	7.62325
names (13,2)= {'p30s3x3brf300RAN13y50'};	2	11	1	0.711	2	17	401	1267	23346.56	9.515	1683	3.83053	9.60	6.90149
names (13,3)= {'p30s3x3brf300REG13clu31x31y85'};	3	11	1	0.721	2	17	396	1551	23504.93	55.684	1775	6.34150	9.48	8.70478
names (13,4)= {'p30s3x3brf300REG13clu28x28y120'};	4	11	1	0.744	2	17	1688	3758	23459.43	155.702	3073	6.48959	41.78	9.81597
names (13,5)= {'p30s3x3brf300REG13clu24x25y100'};	5	11	1	0.776	2	17	1977	3327	23486.53	39.515	3605	6.43505	49.00	8.12822
names (13,6)= {'p30s3x3brf300REG13clu18x19y100'};	6	11	1	0.831	2	17	2340	3200	23247.20	157.732	3557	6.04955	58.08	9.78295
names (13,7)= {'p30s3x3brf300REG13clu12x13y130'};	7	11	1	0.886	2	17	2875	3940	23537.33	24.146	4473	7.72876	71.45	9.09694
names (13,8)= {'p30s3x3brf300REG13clu9x10y120'};	8	11	1	0.913	2	17	3106	4022	23232.45	223.000	4218	8.77240	77.23	8.47487
names (13,9)= {'p30s3x3brf300REG13clu7x7y125'};	9	11	1	0.936	2	17	3471	4221	23327.88	191.116	4564	9.77503	86.35	8.75354
names (13,10)= {'p30s3x3brf300REG13clu4x4y150'};	10	11	1	0.963	2	17	4665	5420	22968.97	276.720	5548	10.67622	116.20	6.30853
names (13,11)= {'p30s3x3brf300REG13clu2x2y180'};	11	11	1	0.982	2	17	6092	6430	21966.07	903.801	6529	12.17637	151.88	6.45984

names (13,12)= {'p30s3x3brf300CLU13y250'};	12	11	1	0.991	2	17	7931	9241	23527.87	24.887	9634	14.16465	197.85	5.89309
names (14,1)= {'p30s3x3brf300REG14y50'};	1	12	1	0.66	2	16	324	1169	23637.56	27.981	1660	3.31331	7.70	8.61164
names (14,2)= {'p30s3x3brf300RAN14y50'};	2	12	1	0.711	2	16	402	1366	23371.45	11.029	1717	6.40070	9.65	8.03053
names (14,3)= {'p30s3x3brf300REG14clu31x31y85'};	3	12	1	0.721	2	16	1706	2503	23463.59	1.609	3195	6.10611	42.25	8.83958
names (14,4)= {'p30s3x3brf300REG14clu28x28y120'};	4	12	1	0.744	2	16	1859	3609	23753.14	88.758	3599	5.14466	46.08	9.75689
names (14,5)= {'p30s3x3brf300REG14clu24x25y100'};	5	12	1	0.776	2	16	1835	3196	23602.95	121.823	3272	5.89279	45.48	6.70400
names (14,6)= {'p30s3x3brf300REG14clu18x19y100'};	6	12	1	0.831	2	16	2593	3203	23136.57	378.615	3475	5.93828	64.43	9.14768
names (14,7)= {'p30s3x3brf300REG14clu12x13y130'};	7	12	1	0.886	2	16	2711	3662	23359.48	36.733	4130	6.99556	67.38	7.58847
names (14,8)= {'p30s3x3brf300REG14clu9x10y120'};	8	12	1	0.913	2	16	3126	3997	23408.26	191.138	4300	8.51612	77.75	9.03478
names (14,9)= {'p30s3x3brf300REG14clu7x7y125'};	9	12	1	0.936	2	16	3094	4276	23417.97	132.204	4355	9.25947	76.95	6.37934
names (14,10)= {'p30s3x3brf300REG14clu4x4y150'};	10	12	1	0.963	2	16	4137	5370	23534.43	28.646	5717	10.53131	103.03	7.82305
names (14,11)= {'p30s3x3brf300REG14clu2x2y180'};	11	12	1	0.982	2	16	5406	6504	23303.34	83.809	6719	11.20855	134.75	6.54606
names (14,12)= {'p30s3x3brf300CLU14y250'};	12	12	1	0.991	2	16	7703	9087	23612.17	148.603	9168	12.10867	192.18	8.38610
names (15,1)= {'p30s3x3brf300REG15y50'};	1	13	1	0.66	2	14	360	925	23492.32	9.667	1628	5.61629	8.65	8.67424
names (15,2)= {'p30s3x3brf300RAN15y50'};	2	13	1	0.711	2	14	399	1308	23388.67	19.400	1699	7.08935	9.63	7.50541
names (15,3)= {'p30s3x3brf300REG15clu31x31y85'};	3	13	1	0.721	2	14	222	1757	23591.30	17.867	2050	6.29677	5.20	7.10167
names (15,4)= {'p30s3x3brf300REG15clu28x28y120'};	4	13	1	0.744	2	14	1867	2929	23536.88	39.922	3436	5.76362	46.33	10.00695
names (15,5)= {'p30s3x3brf300REG15clu24x25y100'};	5	13	1	0.776	2	14	2025	3267	23594.94	39.364	3640	7.18689	50.28	8.35994
names (15,6)= {'p30s3x3brf300REG15clu18x19y100'};	6	13	1	0.831	2	14	2334	3216	23447.65	233.444	3442	6.47369	58.00	8.29282
names (15,7)= {'p30s3x3brf300REG15clu12x13y130'};	7	13	1	0.886	2	14	2794	4332	23678.41	96.675	4454	7.47923	69.50	10.11590
names (15,8)= {'p30s3x3brf300REG15clu9x10y120'};	8	13	1	0.913	2	14	2970	4026	23356.94	45.260	4463	8.32127	73.90	8.47965
names (15,9)= {'p30s3x3brf300REG15clu7x7y125'};	9	13	1	0.936	2	14	3364	4212	23180.07	21.058	4787	10.36710	83.75	8.02923
names (15,10)= {'p30s3x3brf300REG15clu4x4y150'};	10	13	1	0.963	2	14	4566	5399	23230.18	255.745	5516	10.03554	113.80	6.80642
names (15,11)= {'p30s3x3brf300REG15clu2x2y180'};	11	13	1	0.982	2	14	5567	6456	23333.66	190.794	6637	12.67132	138.83	6.77600
names (15,12)= {'p30s3x3brf300CLU15y250'};	12	13	1	0.991	2	14	8037	9272	23475.82	54.573	9541	11.48416	200.58	5.24013
names (16,1)= {'p30s3x3brf300REG16y50'};	1	14	1	0.66	2	16	337	967	23478.39	79.045	1430	6.32698	8.03	8.85389
names (16,2)= {'p30s3x3brf300RAN16y50'};	2	14	1	0.711	2	16	398	1392	23578.13	52.468	1610	5.84392	9.55	7.57981
names (16,3)= {'p30s3x3brf300REG16clu31x31y85'};	3	14	1	0.721	2	16	383	1201	23504.36	13.619	3365	6.55833	9.18	8.37800
names (16,4)= {'p30s3x3brf300REG16clu28x28y120'};	4	14	1	0.744	2	16	1658	3298	23678.92	25.088	3498	5.98289	41.05	6.92312
names (16,5)= {'p30s3x3brf300REG16clu24x25y100'};	5	14	1	0.776	2	16	2112	3222	23534.99	87.658	3564	6.94031	52.40	9.10826
names (16,6)= {'p30s3x3brf300REG16clu18x19y100'};	6	14	1	0.831	2	16	2229	3195	23380.23	224.483	3403	6.42299	55.33	7.95953
names (16,7)= {'p30s3x3brf300REG16clu12x13y130'};	7	14	1	0.886	2	16	2635	4440	23723.19	3.183	5009	8.03032	65.48	10.38644
names (16,8)= {'p30s3x3brf300REG16clu9x10y120'};	8	14	1	0.913	2	16	2989	4049	23387.44	31.305	4523	9.13011	74.33	8.90092
names (16,9)= {'p30s3x3brf300REG16clu7x7y125'};	9	14	1	0.936	2	16	3429	4221	23268.90	114.394	4636	9.82120	85.33	9.39601
names (16,10)= {'p30s3x3brf300REG16clu4x4y150'};	10	14	1	0.963	2	16	4078	5430	23547.00	19.608	5709	9.59838	101.55	5.85895
names (16,11)= {'p30s3x3brf300REG16clu2x2y180'};	11	14	1	0.982	2	16	5213	6385	23488.64	82.706	6674	10.76538	129.93	9.12661
names (16,12)= {'p30s3x3brf300CLU16y250'};	12	14	1	0.991	2	16	7326	9093	23606.30	75.452	9058	13.05997	182.75	8.06804
names (17,1)= {'p30s3x3brf300REG17y50'};	1	15	1	0.66	2	16	349	1403	23325.91	129.803	1318	5.46127	8.33	6.17521
names (17,2)= {'p30s3x3brf300RAN17y50'};	2	15	1	0.711	2	16	402	1133	23452.61	45.771	1434	6.43562	9.65	7.40479
names (17,3)= {'p30s3x3brf300REG17clu31x31y85'};	3	15	1	0.721	2	16	1718	2784	23473.18	67.893	2935	5.96657	42.55	7.50741
names (17,4)= {'p30s3x3brf300REG17clu28x28y120'};	4	15	1	0.744	2	16	1805	3964	23648.74	2.140	4377	5.98700	44.73	9.40422
names (17,5)= {'p30s3x3brf300REG17clu24x25y100'};	5	15	1	0.776	2	16	2010	2994	23429.21	0.095	3894	6.02206	49.85	8.71441
names (17,6)= {'p30s3x3brf300REG17clu18x19y100'};	6	15	1	0.831	2	16	2357	3233	23360.01	35.452	3734	7.33633	58.53	7.38325
names (17,7)= {'p30s3x3brf300REG17clu12x13y130'};	7	15	1	0.886	2	16	2717	4606	23558.96	14.579	4599	7.40073	67.53	6.72356
names (17,8)= {'p30s3x3brf300REG17clu9x10y120'};	8	15	1	0.913	2	16	3074	4028	23417.26	20.919	4576	8.52885	76.45	8.78189
names (17,9)= {'p30s3x3brf300REG17clu7x7y125'};	9	15	1	0.936	2	16	3264	4256	23461.18	86.358	4603	8.72513	81.20	9.01317
names (17,10)= {'p30s3x3brf300REG17clu4x4y150'};	10	15	1	0.963	2	16	4179	5367	23310.18	127.000	5424	10.66630	104.08	6.18733
names (17,11)= {'p30s3x3brf300REG17clu2x2y180'};	11	15	1	0.982	2	16	5360	6432	23569.13	22.278	6933	11.81855	133.60	8.96314
names (17,12)= {'p30s3x3brf300CLU17y250'};	12	15	1	0.991	2	16	8233	9171	23247.54	152.298	9463	11.88887	205.43	6.19959
names (24,1)= {'p30s3x3brf300REG24y50'};	1	16	1	0.66	2	17	338	1354	23425.80	199.221	1249	5.79299	8.03	6.95488
names (24,2)= {'p30s3x3brf300RAN24y50'};	2	16	1	0.711	2	17	403	1469	23582.68	10.753	1815	6.46423	9.65	7.79766
names (24,3)= {'p30s3x3brf300REG24clu31x31y85'};	3	16	1	0.721	2	17	1641	2676	23479.41	65.621	2897	6.85328	40.60	8.12407
names (24,4)= {'p30s3x3brf300REG24clu28x28y120'};	4	16	1	0.744	2	17	1751	2830	23494.60	44.775	3253	6.16732	43.35	7.61183
names (24,5)= {'p30s3x3brf300REG24clu24x25y100'};	5	16	1	0.776	2	17	1900	3230	23507.15	7.134	3765	6.71059	47.08	8.32198
names (24,6)= {'p30s3x3brf300REG24clu18x19y100'};	6	16	1	0.831	2	17	2327	3248	23364.76	76.230	3649	6.01582	57.75	8.41240
names (24,7)= {'p30s3x3brf300REG24clu12x13y130'};	7	16	1	0.886	2	17	2786	4654	23457.38	0.412	5081	7.26354	69.23	7.80790
names (24,8)= {'p30s3x3brf300REG24clu9x10y120'};	8	16	1	0.913	2	17	3236	4074	23282.19	100.630	4429	8.48743	80.48	7.98102
names (24,9)= {'p30s3x3brf300REG24clu7x7y125'};	9	16	1	0.936	2	17	3414	4175	23216.94	341.404	4386	9.67389	84.93	8.29557
names (24,10)= {'p30s3x3brf300REG24clu4x4y150'};	10	16	1	0.963	2	17	4396	5360	23299.55	205.948	5490	9.46825	109.48	7.89789
names (24,11)= {'p30s3x3brf300REG24clu2x2y180'};	11	16	1	0.982	2	17	5582	6498	23253.04	54.911	6864	11.50408	139.13	5.91739
names (24,12)= {'p30s3x3brf300CLU24y250'};	12	16	1	0.991	2	17	7742	9214	23640.44	59.997	9423	12.99452	193.13	9.31760
names (3,1)= {'p30s3x3brf300REG03y50'};	1	1	2	0.66	2	3	11	24	0.000173695	0.002987434	82	1.69920	0.2	0.24456
names (3,2)= {'p30s3x3brf300RAN03y50'};	2	1	2	0.711	2	4	11	28	0.000208152	0.003382136	89	2.18696	0.175	0.24254
names (3,3)= {'p30s3x3brf300REG03clu31x31y85'};	3	1	2	0.721	2	4	1429	1441	0.000257199	0.004009848	1500	1.58825	35.625	0.68035
names (3,4)= {'p30s3x3brf300REG03clu28x28y120'};	4	1	2	0.744	2	4	13	30	8.66E-05	0.002193269	91	1.58082	0.225	0.23219
names (3,5)= {'p30s3x3brf300REG03clu24x25y100'};	5	1	2	0.776	2	4	1673	1684	0.000213126	0.003627743	1740	1.82402	41.725	0.46261
names (3,6)= {'p30s3x3brf300REG03clu18x19y100'};	6	1	2	0.831	2	4	2051	2060	0.000282313	0.004378808	2114	1.03170	51.175	0.29149
names (3,7)= {'p30s3x3brf300REG03clu12x13y130'};	7	1	2	0.886	2	4	2489	2496	0.000203377	0.003748768	2551	0.87485	62.125	0.25970
names (3,8)= {'p30s3x3brf300REG03clu9x10y120'};	8	1	2	0.913	2	4	2907	2915	0.000271306	0.004165095	2971	0.93804	72.575	0.36138
names (3,9)= {'p30s3x3brf300REG03clu7x7y125'};	9	1	2	0.936	2	4	3344	3354	0.000287283	0.004103111	3412	0.78192	83.500	0.52097
names (3,10)= {'p30s3x3brf300REG03clu4x4y150'};	10	1	2	0.963	2	4	4075	4082	0.000287908	0.004447418	4136	0.89088	101.775	0.24870
names (3,11)= {'p30s3x3brf300REG03clu2x2y180'};	11	1	2	0.982	2	4	4533	4540	0.000172319	0.00316904	4594	0.80013	113.225	0.29008
names (3,12)= {'p30s3x3brf300CLU03y250'};	12	1	2	0.991	2	4	7435	7443	0.000208151	0.003729066	7499	0.73303	185.775	0.35814
names (4,1)= {'p30s3x3brf300REG04y50'};	1	2	2	0.66	2	3	9	24	0.000252847	0.004084455	89	1.88191	0.150	0.30555
names (4,2)= {'p30s3x3brf300RAN04y50'};	2	2	2	0.711	2	3	12	26	0.000262431	0.004217771	90	1.28504	0.225	0.26676
names (4,3)= {'p30s3x3brf300REG04clu31x31y85'};	3	2	2	0.721	2	4	1401	1413	0.00023551	0.003706984	1469	1.52067	34.925	0.42976
names (4,4)= {'p30s3x3brf300REG04clu28x28y120'};	4	2	2	0.744	2	4	1615	1624	0.000158165	0.003173437	1681	1.66553	40.275	0.56231
names (4,5)= {'p30s3x3brf300REG04clu24x25y100'};	5	2	2	0.776	2	4	1896	1906	0.000244121	0.00393297	1960	2.04349	47.300	0.33187
names (4,6)= {'p30s3x3brf300REG04clu18x19y100'};	6	2	2	0.831	2	4	2003	2010	0.000228028	0.003617297	2064	1.03436	49.975	0.22826
names (4,7)= {'p30s3x3brf300REG04clu12x13y130'};	7	2	2	0.886	2	4	2304	2313	0.000174924	0.003332207	2367	0.93492	57.500	0.36081
names (4,8)= {'p30s3x3brf300REG04clu9x10y120'};	8	2	2	0.913	2	4	2853	2863	0.000254236	0.003960562	2915	0.84621	71.225	0.22638
names (4,9)= {'p30s3x3brf300REG04clu7x7y125'};	9	2	2	0.936	2	4	3231	3243	0.000301019	0.00445045	3297	0.80785	80.675	0.35577
names (4,10)= {'p30s3x3brf300REG04clu4x4y150'};	10	2	2	0.963	2	4	3479	3488	0.000223209	0.003957499	3542	0.79487	86.875	0.40543
names (4,11)= {'p30s3x3brf300REG04clu2x2y180'};	11	2	2	0.982	2	4	5156	5164	0.000278561	0.004415533	5220	0.87756	128.800	0.64419
names (4,12)= {'p30s3x3brf300CLU04y250'};	12	2	2	0.991	2	4	6085	6093	0.000122516	0.002730284	6149	0.68823	152.025	0.29882
names (5,1)= {'p30s3x3brf300REG05y50'};	1	3	2	0.66	2	3	6	26	0.000222208	0.003611428	88	2.29037	0.075	0.29578
names (5,2)= {'p30s3x3brf300RAN05y50'};	2	3	2	0.711	2	4	6	30	0.000220485	0.003543375	91	2.60917	0.05	0.27905
names (5,3)= {'p30s3x3brf300REG05clu31x31y85'};	3	3	2	0.721	2	4	11	30	0.000148958	0.003149543	91	1.89148	0.175	0.26747
names (5,4)= {'p30s3x3brf300REG05clu28x28y120'};	4	3	2	0.744	2	4	1694	1709	0.000155948	0.003067445	1763	1.70621	42.25	0.44630
names (5,5)= {'p30s3x3brf300REG05clu24x25y100'};	5	3	2	0.776	2	4	1687	1695	0.000194195	0.00332897	1750	1.57532	42.075	0.27644
names (5,6)= {'p30s3x3brf300REG05clu18x19y100'};	6	3	2	0.831	2	4	2128	2140	0.000299096	0.00452458	2195	1.17334	53.1	0.44238
names (5,7)= {'p30s3x3brf300REG05clu12x13y130'};	7	3	2	0.886	2	4	2540	2549	0.000205134	0.003723623	2605	1.16489	63.4	0.40374
names (5,8)= {'p30s3x3brf300REG05clu9x10y120'};	8	3	2	0.913	2	4	3061	3070	0.000292708	0.004288555	3126	0.98846	76.425	0.41593
names (5,9)= {'p30s3x3brf300REG05clu7x7y125'};	9	3	2	0.936	2	4	3120	3128	0.00028042	0.004286682	3185	0.87689	77.9	0.45155
names (5,10)= {'p30s3x3brf300REG05clu4x4y150'};	10	3	2	0.963	2	4	3967	3976	0.000231832	0.00370816	4031	0.94647	99.075	0.29091
names (5,11)= {'p30s3x3brf300REG05clu2x2y180'};	11	3	2	0.982	2	4	4887	4895	0.000197551	0.0033641	4950	0.73855	122.075	0.45763
names (5,12)= {'p30s3x3brf300CLU05y250'};	12	3	2	0.991	2	4	7450	7458	0.000184778	0.003299063	7512	0.73227	186.15	0.31804
names (6,1)= {'p30s3x3brf300REG06y75'};	1	4	2	0.66	2	4	7	27	0.000147253	0.002946321	89	2.88685	0.075	0.28756
names (6,2)= {'p30s3x3brf300RAN06y50'};	2	4	2	0.711	2	4	12	25	0.000256709	0.004087536	93	2.46945	0.2	0.29649
names (6,3)= {'p30s3x3brf300REG06clu31x31y85'};	3	4	2	0.721	2	4	1552	1559	0.00025423	0.003862464	1613	2.15602	38.7	0.30028
names (6,4)= {'p30s3x3brf300REG06clu28x28y120'};	4	4	2	0.744	2	4	1500	1507	0.0001634	0.003322337	1565	1.37015	37.4	0.63415
names (6,5)= {'p30s3x3brf300REG06clu24x25y100'};	5	4	2	0.776	2	4	1913	1923	0.000260987	0.00420482	1978	1.22075	47.725	0.43867
names (6,6)= {'p30s3x3brf300REG06clu18x19y100'};	6	4	2	0.831	2	4	1888	1895	0.00024594	0.004002882	1949	1.14408	47.1	0.26683
names (6,7)= {'p30s3x3brf300REG06clu12x13y130'};	7	4	2	0.886	2	4	2277	2283	0.000175902	0.003376149	2340	1.04851	56.825	0.44306
names (6,8)= {'p30s3x3brf300REG06clu9x10y120'};	8	4	2	0.913	2	4	2900	2907	0.000248758	0.003815071	2969	0.90439	72.4	0.79636
names (6,9)= {'p30s3x3brf300REG06clu7x7y125'};	9	4	2	0.936	2	4	3120	3129	0.00025262	0.003863718	3185	0.83665	77.9	0.39027
names (6,10)= {'p30s3x3brf300REG06clu4x4y150'};	10	4	2	0.963	2	4	3692	3700	0.000209192	0.003551565	3756	0.82273	92.2	0.34409
names (6,11)= {'p30s3x3brf300REG06clu2x2y180'};	11	4	2	0.982	2	4	5297	5305	0.000287192	0.004399873	5359	0.88713	132.325	0.39951
names (6,12)= {'p30s3x3brf300CLU06y250'};	12	4	2	0.991	2	4	6701	6708	0.000138136	0.002828195	6763	0.81808	167.425	0.29038
names (7,1)= {'p30s3x3brf300REG07y50'};	1	5	2	0.66	2	4	8	31	0.000228927	0.003672069	92	2.57931	0.1	0.27799
names (7,2)= {'p30s3x3brf300RAN07y50'};	2	5	2	0.711	2	4	9	25	0.000242755	0.003960209	86	2.61910	0.125	0.25091
names (7,3)= {'p30s3x3brf300REG07clu31x31y85'};	3	5	2	0.721	2	4	11	30	0.000147171	0.003124573	89	2.40096	0.175	0.22956
names (7,4)= {'p30s3x3brf300REG07clu28x28y120'};	4	5	2	0.744	2	4	1698	1708	0.000150952	0.002984852	1761	1.85478	42.35	0.27127
names (7,5)= {'p30s3x3brf300REG07clu24x25y100'};	5	5	2	0.776	2	4	1695	1702	0.000207107	0.003517944	1756	1.44209	42.275	0.23606
names (7,6)= {'p30s3x3brf300REG07clu18x19y100'};	6	5	2	0.831	2	4	2211	2221	0.000300824	0.004452063	2278	1.20777	55.175	0.42355
names (7,7)= {'p30s3x3brf300REG07clu12x13y130'};	7	5	2	0.886	2	4	2308	2315	0.000159426	0.003052994	2371	1.04333	57.6	0.29469
names (7,8)= {'p30s3x3brf300REG07clu9x10y120'};	8	5	2	0.913	2	4	2844	2855	0.000292287	0.004525546	2915	0.90319	71	0.94623
names (7,9)= {'p30s3x3brf300REG07clu7x7y125'};	9	5	2	0.936	2	4	2976	2986	0.000241482	0.003823259	3042	0.88287	74.3	0.39591
names (7,10)= {'p30s3x3brf300REG07clu4x4y150'};	10	5	2	0.963	2	4	3704	3712	0.000212202	0.003583117	3768	0.79944	92.5	0.48755
names (7,11)= {'p30s3x3brf300REG07clu2x2y180'};	11	5	2	0.982	2	4	5575	5584	0.000286695	0.004047637	5637	0.82504	139.275	0.37913
names (7,12)= {'p30s3x3brf300CLU07y250'};	12	5	2	0.991	2	4	8060	8067	0.000262692	0.004078033	8121	0.78385	201.4	0.24902
names (8,1)= {'p30s3x3brf300REG08y50'};	1	6	2	0.66	2	3	8	26	0.000212408	0.003450134	89	2.10815	0.125	0.24456
names (8,2)= {'p30s3x3brf300RAN08y50'};	2	6	2	0.711	2	4	10	27	0.000255777	0.004116871	90	3.09314	0.15	0.27818
names (8,3)= {'p30s3x3brf300REG08clu31x31y85'};	3	6	2	0.721	2	4	1585	1592	0.000256797	0.003871467	1649	1.93516	39.525	0.30992
names (8,4)= {'p30s3x3brf300REG08clu28x28y120'};	4	6	2	0.744	2	4	1508	1516	0.000156913	0.003204229	1571	1.78438	37.6	0.38792
names (8,5)= {'p30s3x3brf300REG08clu24x25y100'};	5	6	2	0.776	2	4	1864	1870	0.00024205	0.003959127	1924	1.85565	46.5	0.26540
names (8,6)= {'p30s3x3brf300REG08clu18x19y100'};	6	6	2	0.831	2	4	1953	1961	0.000281871	0.004488348	2018	1.29521	48.725	0.49916
names (8,7)= {'p30s3x3brf300REG08clu12x13y130'};	7	6	2	0.886	2	4	2689	2700	0.000223958	0.003959004	2753	1.08086	67.125	0.31896
names (8,8)= {'p30s3x3brf300REG08clu9x10y120'};	8	6	2	0.913	2	4	2642	2652	0.000230808	0.003779777	2706	1.03768	65.95	0.36950
names (8,9)= {'p30s3x3brf300REG08clu7x7y125'};	9	6	2	0.936	2	4	3055	3066	0.00026669	0.004123307	3121	0.88342	76.275	0.38362
names (8,10)= {'p30s3x3brf300REG08clu4x4y150'};	10	6	2	0.963	2	4	3802	3809	0.000241049	0.00398289	3865	0.84910	94.95	0.47539
names (8,11)= {'p30s3x3brf300REG08clu2x2y180'};	11	6	2	0.982	2	4	5253	5265	0.000263217	0.004066671	5322	0.87959	131.225	0.51385
names (8,12)= {'p30s3x3brf300CLU08y250'};	12	6	2	0.991	2	4	6572	6583	0.000135479	0.002816826	6641	0.69973	164.2	0.78682

names (9,1)= {'p30s3x3brf300REG09y50'};	1	7	2	0.66	2	4	7	24	0.000227327	0.003688011	88	3.77149	0.075	0.29886
names (9,2)= {'p30s3x3brf300RAN09y50'};	2	7	2	0.711	2	4	9	25	0.000205242	0.003358834	87	2.68699	0.125	0.26338
names (9,3)= {'p30s3x3brf300REG09clu31x31y85'};	3	7	2	0.721	2	4	1418	1429	0.000231532	0.003637291	1486	1.53414	35.35	0.48818
names (9,4)= {'p30s3x3brf300REG09clu28x28y120'};	4	7	2	0.744	2	4	1577	1585	0.000161166	0.003236917	1644	1.79062	39.325	1.04769
names (9,5)= {'p30s3x3brf300REG09clu24x25y100'};	5	7	2	0.776	2	4	1740	1748	0.000210196	0.003542455	1803	1.35906	43.4	0.34499
names (9,6)= {'p30s3x3brf300REG09clu18x19y100'};	6	7	2	0.831	2	4	2164	2174	0.000311608	0.004681904	2228	1.41130	54	0.40715
names (9,7)= {'p30s3x3brf300REG09clu12x13y130'};	7	7	2	0.886	2	4	2485	2496	0.000181217	0.003342784	2551	1.06964	62.025	0.29871
names (9,8)= {'p30s3x3brf300REG09clu9x10y120'};	8	7	2	0.913	2	4	2851	2864	0.000248816	0.003847824	2921	0.91372	71.175	0.93311
names (9,9)= {'p30s3x3brf300REG09clu7x7y125'};	9	7	2	0.936	2	4	2753	2763	0.000257695	0.004293736	2823	0.83431	68.725	0.64096
names (9,10)= {'p30s3x3brf300REG09clu4x4y150'};	10	7	2	0.963	2	4	3924	3938	0.000236117	0.003776028	3993	0.72347	98	0.41400
names (9,11)= {'p30s3x3brf300REG09clu2x2y180'};	11	7	2	0.982	2	4	4751	4760	0.000228117	0.003971662	4812	0.74627	118.675	0.27193
names (9,12)= {'p30s3x3brf300CLU09y250'};	12	7	2	0.991	2	4	6674	6681	0.000169027	0.00344754	6734	0.75340	166.75	0.26725
names (10,1)= {'p30s3x3brf300REG10y50'};	1	8	2	0.66	2	4	10	19	0.000206504	0.003378168	86	3.52393	0.15	0.28350
names (10,2)= {'p30s3x3brf300RAN10y50'};	2	8	2	0.711	2	4	11	25	0.000208905	0.003383225	90	2.26088	0.175	0.28174
names (10,3)= {'p30s3x3brf300REG10clu31x31y85'};	3	8	2	0.721	2	4	1367	1377	0.000252912	0.004011343	1433	1.84029	34.075	0.35352
names (10,4)= {'p30s3x3brf300REG10clu28x28y120'};	4	8	2	0.744	2	4	1581	1591	0.00014976	0.003028118	1646	2.06085	39.425	0.42878
names (10,5)= {'p30s3x3brf300REG10clu24x25y100'};	5	8	2	0.776	2	4	1590	1596	0.000195337	0.003401956	1654	1.87758	39.65	0.34854
names (10,6)= {'p30s3x3brf300REG10clu18x19y100'};	6	8	2	0.831	2	4	1906	1915	0.000224784	0.003641216	1970	1.22109	47.55	0.32497
names (10,7)= {'p30s3x3brf300REG10clu12x13y130'};	7	8	2	0.886	2	4	2617	2625	0.000186585	0.003359078	2681	1.17894	65.325	0.35578
names (10,8)= {'p30s3x3brf300REG10clu9x10y120'};	8	8	2	0.913	2	4	2560	2567	0.00020335	0.003412182	2625	1.00118	63.9	0.43428
names (10,9)= {'p30s3x3brf300REG10clu7x7y125'};	9	8	2	0.936	2	4	3076	3083	0.000273783	0.004242157	3139	1.01856	76.8	0.33895
names (10,10)= {'p30s3x3brf300REG10clu4x4y150'};	10	8	2	0.963	2	4	3509	3516	0.000214401	0.003782486	3570	0.87932	87.625	0.37820
names (10,11)= {'p30s3x3brf300REG10clu2x2y180'};	11	8	2	0.982	2	4	5103	5111	0.000247273	0.003975754	5167	0.78354	127.475	0.40651
names (10,12)= {'p30s3x3brf300CLU10y250'};	12	8	2	0.991	2	4	7222	7231	0.000171722	0.003193416	7286	0.77192	180.45	0.45143
names (11,1)= {'p30s3x3brf300REG11y50'};	1	9	2	0.66	2	4	8	25	0.000241816	0.003929989	87	2.67543	0.1	0.27277
names (11,2)= {'p30s3x3brf300RAN11y50'};	2	9	2	0.711	2	4	10	26	0.000222017	0.003624457	87	2.34377	0.15	0.24725
names (11,3)= {'p30s3x3brf300REG11clu31x31y85'};	3	9	2	0.721	2	4	1616	1623	0.000319214	0.004737146	1679	1.84571	40.3	0.33201
names (11,4)= {'p30s3x3brf300REG11clu28x28y120'};	4	9	2	0.744	2	4	1624	1631	0.000172117	0.003443066	1685	1.69303	40.5	0.27021
names (11,5)= {'p30s3x3brf300REG11clu24x25y100'};	5	9	2	0.776	2	4	1741	1750	0.000228024	0.003832309	1805	2.02667	43.425	0.34287
names (11,6)= {'p30s3x3brf300REG11clu18x19y100'};	6	9	2	0.831	2	4	2046	2056	0.000261809	0.004070074	2110	1.44522	51.05	0.40207
names (11,7)= {'p30s3x3brf300REG11clu12x13y130'};	7	9	2	0.886	2	4	2598	2606	0.000175654	0.00318203	2663	0.90754	64.85	0.37352
names (11,8)= {'p30s3x3brf300REG11clu9x10y120'};	8	9	2	0.913	2	4	2629	2640	0.00024039	0.003941108	2694	0.83483	65.625	0.31184
names (11,9)= {'p30s3x3brf300REG11clu7x7y125'};	9	9	2	0.936	2	4	2875	2885	0.00025061	0.004061905	2940	0.94872	71.775	0.51107
names (11,10)= {'p30s3x3brf300REG11clu4x4y150'};	10	9	2	0.963	2	4	3482	3495	0.000221326	0.003899029	3550	0.74720	86.95	0.50205
names (11,11)= {'p30s3x3brf300REG11clu2x2y180'};	11	9	2	0.982	2	4	5445	5453	0.000300544	0.004412549	5506	0.75810	136.025	0.39407
names (11,12)= {'p30s3x3brf300CLU11y250'};	12	9	2	0.991	2	4	6538	6546	0.000154739	0.003217052	6602	0.61210	163.35	0.43972
names (12,1)= {'p30s3x3brf300REG12y50'};	1	10	2	0.66	2	4	6	28	0.000231105	0.003727271	89	3.40596	0.05	0.26887
names (12,2)= {'p30s3x3brf300RAN12y50'};	2	10	2	0.711	2	4	11	25	0.00022669	0.003665006	90	1.64097	0.175	0.28246
names (12,3)= {'p30s3x3brf300REG12clu31x31y85'};	3	10	2	0.721	2	4	11	34	0.000138074	0.00288789	95	1.82141	0.175	0.26848
names (12,4)= {'p30s3x3brf300REG12clu28x28y120'};	4	10	2	0.744	2	4	13	37	0.000114838	0.002850116	94	1.26659	0.225	0.23391
names (12,5)= {'p30s3x3brf300REG12clu24x25y100'};	5	10	2	0.776	2	4	1732	1741	0.000233727	0.003932458	1794	1.28809	43.2	0.25974
names (12,6)= {'p30s3x3brf300REG12clu18x19y100'};	6	10	2	0.831	2	4	1828	1835	0.000220257	0.003649674	1890	0.94934	45.6	0.26527
names (12,7)= {'p30s3x3brf300REG12clu12x13y130'};	7	10	2	0.886	2	4	2560	2569	0.000190526	0.003463942	2625	0.95239	63.9	0.42585
names (12,8)= {'p30s3x3brf300REG12clu9x10y120'};	8	10	2	0.913	2	4	2724	2733	0.000236857	0.003808453	2788	1.08042	68	0.36077
names (12,9)= {'p30s3x3brf300REG12clu7x7y125'};	9	10	2	0.936	2	4	2929	2937	0.000249211	0.003997802	2992	0.90652	73.125	0.41849
names (12,10)= {'p30s3x3brf300REG12clu4x4y150'};	10	10	2	0.963	2	4	3876	3888	0.000257571	0.004160618	3945	0.84888	96.8	0.57494
names (12,11)= {'p30s3x3brf300REG12clu2x2y180'};	11	10	2	0.982	2	4	4812	4823	0.000232524	0.003999707	4877	0.79102	120.2	0.39241
names (12,12)= {'p30s3x3brf300CLU12y250'};	12	10	2	0.991	2	4	7890	7897	0.000259649	0.004182593	7957	0.68823	197.15	0.74092
names (13,1)= {'p30s3x3brf300REG13y50'};	1	11	2	0.66	2	4	10	25	0.000240319	0.003916391	87	2.46018	0.15	0.26980
names (13,2)= {'p30s3x3brf300RAN13y50'};	2	11	2	0.711	2	4	10	27	0.000201369	0.003283976	88	1.68987	0.15	0.23941
names (13,3)= {'p30s3x3brf300REG13clu31x31y85'};	3	11	2	0.721	2	4	14	29	0.000153385	0.00322277	92	1.92652	0.25	0.24415
names (13,4)= {'p30s3x3brf300REG13clu28x28y120'};	4	11	2	0.744	2	4	1462	1471	0.000151363	0.003099605	1530	1.88940	36.45	0.46819
names (13,5)= {'p30s3x3brf300REG13clu24x25y100'};	5	11	2	0.776	2	4	1735	1745	0.000220026	0.003693413	1801	1.43271	43.275	0.42893
names (13,6)= {'p30s3x3brf300REG13clu18x19y100'};	6	11	2	0.831	2	4	2099	2105	0.000298848	0.004586414	2160	1.11929	52.375	0.35783
names (13,7)= {'p30s3x3brf300REG13clu12x13y130'};	7	11	2	0.886	2	4	2597	2606	0.000211807	0.003822247	2660	1.02358	64.825	0.33364
names (13,8)= {'p30s3x3brf300REG13clu9x10y120'};	8	11	2	0.913	2	4	2815	2824	0.000272464	0.004273895	2879	0.82504	70.275	0.33842
names (13,9)= {'p30s3x3brf300REG13clu7x7y125'};	9	11	2	0.936	2	4	3160	3169	0.00030116	0.004534166	3225	0.78688	78.9	0.53777
names (13,10)= {'p30s3x3brf300REG13clu4x4y150'};	10	11	2	0.963	2	4	4295	4302	0.000331465	0.004802618	4359	0.83270	107.275	0.56624
names (13,11)= {'p30s3x3brf300REG13clu2x2y180'};	11	11	2	0.982	2	4	5721	5729	0.000391229	0.005269936	5780	0.94951	142.925	0.16661
names (13,12)= {'p30s3x3brf300CLU13y250'};	12	11	2	0.991	2	4	7489	7497	0.000215142	0.003805779	7550	0.75481	187.125	0.27791
names (14,1)= {'p30s3x3brf300REG14y50'};	1	12	2	0.66	2	4	11	25	0.000231158	0.003755013	87	3.57175	0.175	0.24600
names (14,2)= {'p30s3x3brf300RAN14y50'};	2	12	2	0.711	2	4	14	29	0.000259048	0.004139969	92	1.92960	0.25	0.26062
names (14,3)= {'p30s3x3brf300REG14clu31x31y85'};	3	12	2	0.721	2	4	1449	1459	0.000267544	0.004158801	1514	1.48681	36.125	0.39152
names (14,4)= {'p30s3x3brf300REG14clu28x28y120'};	4	12	2	0.744	2	4	1619	1628	0.000165699	0.003297578	1681	1.47046	40.375	0.36322
names (14,5)= {'p30s3x3brf300REG14clu24x25y100'};	5	12	2	0.776	2	4	1612	1620	0.000197663	0.00343304	1675	1.49808	40.2	0.36027
names (14,6)= {'p30s3x3brf300REG14clu18x19y100'};	6	12	2	0.831	2	4	2313	2320	0.000301678	0.004375738	2374	1.19776	57.725	0.22577
names (14,7)= {'p30s3x3brf300REG14clu12x13y130'};	7	12	2	0.886	2	4	2413	2421	0.000186299	0.003483349	2479	1.36355	60.225	0.45872
names (14,8)= {'p30s3x3brf300REG14clu9x10y120'};	8	12	2	0.913	2	4	2816	2828	0.000277522	0.004332583	2883	0.91369	70.3	0.56165
names (14,9)= {'p30s3x3brf300REG14clu7x7y125'};	9	12	2	0.936	2	4	2773	2781	0.000223052	0.00373317	2836	0.91983	69.225	0.28967
names (14,10)= {'p30s3x3brf300REG14clu4x4y150'};	10	12	2	0.963	2	4	3801	3810	0.000238113	0.003941884	3864	0.79126	94.925	0.30828
names (14,11)= {'p30s3x3brf300REG14clu2x2y180'};	11	12	2	0.982	2	4	5025	5033	0.000249788	0.004099178	5089	0.82489	125.525	0.43512
names (14,12)= {'p30s3x3brf300CLU14y250'};	12	12	2	0.991	2	4	7256	7262	0.000196979	0.003648952	7319	0.78423	181.3	0.42241
names (15,1)= {'p30s3x3brf300REG15y50'};	1	13	2	0.66	2	4	9	28	0.000234013	0.003788793	89	2.57610	0.125	0.26649
names (15,2)= {'p30s3x3brf300RAN15y50'};	2	13	2	0.711	2	4	8	25	0.000204883	0.003335342	88	2.52842	0.1	0.26586
names (15,3)= {'p30s3x3brf300REG15clu31x31y85'};	3	13	2	0.721	2	4	13	32	0.000153362	0.003239342	91	1.54950	0.225	0.24232
names (15,4)= {'p30s3x3brf300REG15clu28x28y120'};	4	13	2	0.744	2	4	1638	1648	0.000168538	0.003361136	1702	2.05697	40.85	0.31287
names (15,5)= {'p30s3x3brf300REG15clu24x25y100'};	5	13	2	0.776	2	4	1774	1782	0.000236332	0.003948613	1837	1.85282	44.25	0.32920
names (15,6)= {'p30s3x3brf300REG15clu18x19y100'};	6	13	2	0.831	2	4	2045	2054	0.000283288	0.004404574	2111	1.26635	51.025	0.46002
names (15,7)= {'p30s3x3brf300REG15clu12x13y130'};	7	13	2	0.886	2	4	2504	2511	0.000188289	0.003462449	2565	0.94952	62.5	0.24979
names (15,8)= {'p30s3x3brf300REG15clu9x10y120'};	8	13	2	0.913	2	4	2669	2679	0.000217399	0.003550902	2734	1.01677	66.625	0.38012
names (15,9)= {'p30s3x3brf300REG15clu7x7y125'};	9	13	2	0.936	2	4	3055	3061	0.000255173	0.003976189	3121	1.16309	76.275	0.42504
names (15,10)= {'p30s3x3brf300REG15clu4x4y150'};	10	13	2	0.963	2	4	4192	4203	0.000294692	0.004392421	4258	0.74285	104.7	0.41390
names (15,11)= {'p30s3x3brf300REG15clu2x2y180'};	11	13	2	0.982	2	4	5146	5153	0.000226647	0.003627238	5210	0.76164	128.55	0.48139
names (15,12)= {'p30s3x3brf300CLU15y250'};	12	13	2	0.991	2	4	7595	7605	0.000222425	0.003830073	7659	0.72757	189.775	0.45749
names (16,1)= {'p30s3x3brf300REG16y50'};	1	14	2	0.66	2	4	11	24	0.000207076	0.003390087	86	2.57009	0.175	0.23763
names (16,2)= {'p30s3x3brf300RAN16y50'};	2	14	2	0.711	2	4	12	23	0.000205329	0.003351122	88	1.70973	0.2	0.25586
names (16,3)= {'p30s3x3brf300REG16clu31x31y85'};	3	14	2	0.721	2	4	12	28	0.000133815	0.002848664	90	1.74256	0.2	0.25024
names (16,4)= {'p30s3x3brf300REG16clu28x28y120'};	4	14	2	0.744	2	4	1441	1448	0.000134097	0.002774257	1502	2.03820	35.925	0.23671
names (16,5)= {'p30s3x3brf300REG16clu24x25y100'};	5	14	2	0.776	2	4	1880	1889	0.000272301	0.004414447	1945	1.35001	46.9	0.44739
names (16,6)= {'p30s3x3brf300REG16clu18x19y100'};	6	14	2	0.831	2	4	1983	1992	0.000252583	0.003977376	2048	1.14140	49.475	0.47286
names (16,7)= {'p30s3x3brf300REG16clu12x13y130'};	7	14	2	0.886	2	4	2369	2377	0.00016745	0.003167204	2431	0.82643	59.125	0.32709
names (16,8)= {'p30s3x3brf300REG16clu9x10y120'};	8	14	2	0.913	2	4	2717	2728	0.000251109	0.004033619	2782	1.08648	67.825	0.31669
names (16,9)= {'p30s3x3brf300REG16clu7x7y125'};	9	14	2	0.936	2	4	3119	3128	0.000298022	0.004526895	3183	0.79823	77.875	0.48396
names (16,10)= {'p30s3x3brf300REG16clu4x4y150'};	10	14	2	0.963	2	4	3742	3751	0.000237299	0.00398286	3808	1.07874	93.45	0.49062
names (16,11)= {'p30s3x3brf300REG16clu2x2y180'};	11	14	2	0.982	2	4	4804	4812	0.000232896	0.004013294	4866	0.86125	120	0.36282
names (16,12)= {'p30s3x3brf300CLU16y250'};	12	14	2	0.991	2	4	6912	6918	0.000179408	0.003526382	6971	0.80476	172.7	0.22200
names (17,1)= {'p30s3x3brf300REG17y50'};	1	15	2	0.66	2	4	8	23	0.000238955	0.003858059	89	2.45563	0.1	0.30691
names (17,2)= {'p30s3x3brf300RAN17y50'};	2	15	2	0.711	2	4	10	27	0.000255382	0.004125276	88	1.90662	0.15	0.24958
names (17,3)= {'p30s3x3brf300REG17clu31x31y85'};	3	15	2	0.721	2	4	1434	1443	0.000245479	0.003860959	1498	2.01492	35.75	0.30222
names (17,4)= {'p30s3x3brf300REG17clu28x28y120'};	4	15	2	0.744	2	4	1572	1579	0.00014893	0.003006281	1635	1.65563	39.2	0.32987
names (17,5)= {'p30s3x3brf300REG17clu24x25y100'};	5	15	2	0.776	2	4	1775	1781	0.000250719	0.004174241	1838	1.07441	44.275	0.48874
names (17,6)= {'p30s3x3brf300REG17clu18x19y100'};	6	15	2	0.831	2	4	2093	2101	0.0002618	0.004036895	2158	1.16713	52.225	0.45198
names (17,7)= {'p30s3x3brf300REG17clu12x13y130'};	7	15	2	0.886	2	4	2428	2435	0.000191545	0.003571198	2492	1.05122	60.6	0.45779
names (17,8)= {'p30s3x3brf300REG17clu9x10y120'};	8	15	2	0.913	2	4	2776	2787	0.000246872	0.003923599	2841	0.79258	69.3	0.30288
names (17,9)= {'p30s3x3brf300REG17clu7x7y125'};	9	15	2	0.936	2	4	2954	2962	0.000234305	0.003754666	3014	0.95339	73.75	0.19095
names (17,10)= {'p30s3x3brf300REG17clu4x4y150'};	10	15	2	0.963	2	4	3818	3826	0.000218866	0.003607997	3880	0.72462	95.35	0.33569
names (17,11)= {'p30s3x3brf300REG17clu2x2y180'};	11	15	2	0.982	2	4	4955	4971	0.000229109	0.003803325	5029	0.65162	123.775	0.75551
names (17,12)= {'p30s3x3brf300CLU17y250'};	12	15	2	0.991	2	4	7777	7783	0.000205391	0.003431146	7837	0.77125	194.325	0.28055
names (24,1)= {'p30s3x3brf300REG24y50'};	1	16	2	0.66	2	4	7	25	0.000248996	0.004025761	88	2.71523	0.075	0.28646
names (24,2)= {'p30s3x3brf300RAN24y50'};	2	16	2	0.711	2	4	10	26	0.000211962	0.003441008	89	2.53118	0.15	0.26551
names (24,3)= {'p30s3x3brf300REG24clu31x31y85'};	3	16	2	0.721	2	4	1393	1402	0.000267374	0.004209982	1456	2.82683	34.725	0.37269
names (24,4)= {'p30s3x3brf300REG24clu28x28y120'};	4	16	2	0.744	2	4	1513	1522	0.000139869	0.002860508	1576	2.90594	37.725	0.31131
names (24,5)= {'p30s3x3brf300REG24clu24x25y100'};	5	16	2	0.776	2	4	1659	1668	0.000195968	0.003364305	1725	1.45323	41.375	0.36386
names (24,6)= {'p30s3x3brf300REG24clu18x19y100'};	6	16	2	0.831	2	4	2068	2077	0.00028601	0.004419736	2132	1.37408	51.6	0.34007
names (24,7)= {'p30s3x3brf300REG24clu12x13y130'};	7	16	2	0.886	2	4	2489	2501	0.000186784	0.00344496	2557	1.01566	62.125	0.57003
names (24,8)= {'p30s3x3brf300REG24clu9x10y120'};	8	16	2	0.913	2	4	2932	2940	0.000249902	0.003805989	2997	0.95842	73.2	0.41074
names (24,9)= {'p30s3x3brf300REG24clu7x7y125'};	9	16	2	0.936	2	4	3099	3107	0.000266676	0.004108617	3161	1.06312	77.375	0.30095
names (24,10)= {'p30s3x3brf300REG24clu4x4y150'};	10	16	2	0.963	2	4	4044	4052	0.000291013	0.004518966	4105	0.86873	101	0.33540
names (24,11)= {'p30s3x3brf300REG24clu2x2y180'};	11	16	2	0.982	2	4	5144	5158	0.000240071	0.003804465	5224	0.76101	128.5	0.35569
names (24,12)= {'p30s3x3brf300CLU24y250'};	12	16	2	0.991	2	4	7278	7286	0.000194247	0.003557996	7339	0.83601	181.85	0.31943
